# On the direct use of CO_2_ in multicomponent reactions: introducing the Passerini four component reaction†

**DOI:** 10.1039/c8ra07150k

**Published:** 2018-09-07

**Authors:** Kelechukwu Nnabuike Onwukamike, Stéphane Grelier, Etienne Grau, Henri Cramail, Michael A. R. Meier

**Affiliations:** Institute of Organic Chemistry (IOC), Materialwissenschaftliches Zentrum (MZE), Karlsruhe Institute of Technology (KIT) Straße am Forum 7 76131 Karlsruhe Germany m.a.r.meier@kit.edu https://www.meier-michael.com; Univ. Bordeaux, CNRS, Bordeaux INP/ENSCBP, Laboratoire de Chimie des Polymères Organiques, UMR 5629 16 Avenue Pey-Berland, F-33607 Pessac Cedex France cramail@enscbp.fr htpps://www.lcpo.fr; Centre National de la Recherche Scientifique, Laboratoire de Chimie des Polymères Organiques, UMR 5629 16 Avenue Pey-Berland, F-33607 Pessac Cedex France

## Abstract

We introduce a novel isocyanide-based multicomponent reaction, the Passerini four component reaction (P-4CR), by replacing the carboxylic acid component of a conventional Passerini three component reaction (P-3CR) with an alcohol and CO_2_. Key to this approach is the use of a switchable solvent system, allowing the synthesis of a variety of α-carbonate-amides. The reaction was first investigated and optimized using butanol, isobutyraldehyde, *tert*-butyl isocyanide and CO_2_. Parameters investigated included the effect of reactant equivalents, reactant concentration, solvent, catalyst, catalyst concentration and CO_2_ pressure. Of the other parameters, the purity of the aldehyde and its tendency to oxidize was one of the most critical parameters for a successful P-4CR. After optimization, a total of twelve (12) P-4CR compounds were synthesized with conversions ranging between 16 and 82% and isolated yields between 18 and 43%. Their structures were confirmed *via*^1^H and ^13^C NMR, FT-IR and high resolution mass spectrometry (ESI-MS). In addition, three (3) hydrolysis products of P-4CR (α-hydroxyl-amides) were successfully isolated with yields between 23 and 63% and fully characterized (^1^H, ^13^C NMR, FT-IR and ESI-MS) as well.

## Abstract 2

We introduce a novel isocyanide-based multicomponent reaction, the Passerini four component reaction (P-4CR), by replacing the carboxylic acid component of a conventional Passerini three component reaction (P-3CR) with an alcohol and CO_2_.

## Graphical abstract

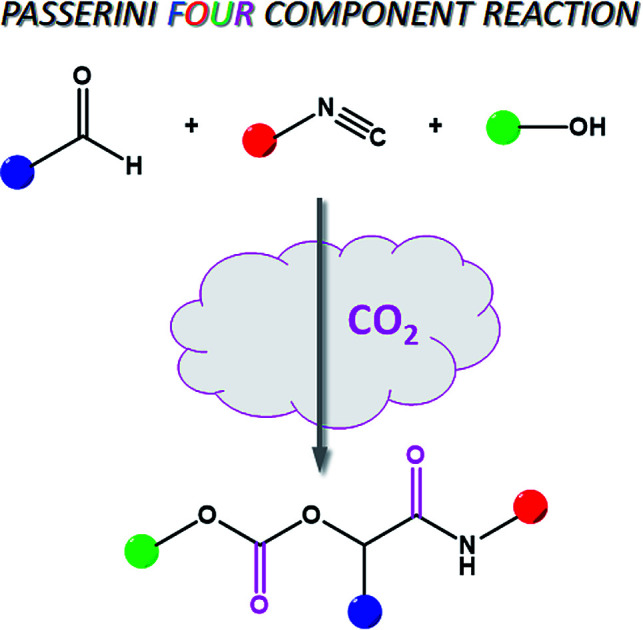

## 1. Introduction

Multicomponent reactions (MCRs) are defined as reactions involving more than two starting materials, while forming products in which most of the atoms of the starting materials are incorporated.^1^ Already in 1850, Strecker reported the synthesis of α-amino-nitriles from an aldehyde, ammonia and hydrogen cyanide, one the first reported MCRs.^2^ Today, a large variety of different types of MCR exist.^3^ In the context of this work, isocyanide-based multicomponent reactions (IMCRs) are of particular interest. Prominent examples of IMCRs are the Passerini three component reaction (P-3CR) and Ugi four component reaction (Ugi 4-CR). The P-3CR was discovered in 1921 by Mario Passerini and describes the reaction between a carboxylic acid, carbonyl component (aldehyde or ketone) and an isocyanide, forming α-acyloxyl-amides.^4^ The Ugi four component reaction (Ugi 4-CR) was developed by Ivar Ugi in 1960 and involves a carboxylic acid, carbonyl component (aldehyde or ketone), an isocyanide and an amine as components, leading to the formation of a bis-amide.^5^ Several variations of the IMCRs exist, one of them being the Ugi 5-CR that uses an alcohol (usually methanol) and CO_2_ as acid component.^6^ This strategy was also employed in combination with efficient thiol–ene polymerization to synthesize highly functionalized polycarbonates, polyamides, polyurethanes and polyhydantoins.^7^ Besides polymer chemistry, MCRs have equally found application in high throughput synthesis,^8^ as well as in combinatorial chemistry,^9^ allowing the synthesis and screening of compound libraries. Furthermore, IMCRs are also very useful in the synthesis of heterocycles.^10^ Most recently, the Ugi 4-CR of perfluorinated acids was employed for the synthesis of a library of molecular keys that were applied for molecular cryptography.^11^

Apart from the classic P-3CR, many variations have been reported. Taguchi and co-workers reported on the direct utilization of aliphatic alcohols alongside an isocyanide and α,β-unsaturated aldehyde in the presence of an Indium(iii) catalyst to form α-alkoxy-amide products.^12^ El Kaim *et al.* employed the so-called Passerini-Smiles reaction of *o*-nitrophenol as a replacement of the acid component and synthesized a library of α-aryloxy-amide products.^13^ Chatani *et al.* reported the reaction of cyclic and acyclic acetals with isocyanides in the presence of GaCl_3_ as catalyst.^14^ Here, the isocyanide inserts into the C–O bond of the acetals, finally resulting in α-alkoxy-imidates. Denmark and Yan reported the asymmetric α-addition of isocyanides to aldehydes. In this case, the reaction was catalyzed by a combination of a weak Lewis acid, SiCl_4_ activated by a chiral Lewis base (bisphosphoramide). The desired α-hydroxyl-amides were obtained after basic workup.^15^

Very important for the herein reported results, Jessop and co-workers introduced switchable solvent systems involving CO_2_ alongside a super base in 2005.^16^ The unique nature of this solvent system was its ability to switch from a non-polar to polar solvent in the absence or presence of CO_2_, respectively, by the reversible formation of a carbonate anion/protonated base complex. The system is very versatile and allows applications such as straight-forward product purification,^17^ as CO_2_ capturing agents,^18^ for the selective extraction of hemicellulose from wood,^19^ and also as sustainable solvents for cellulose solubilization.^20,21^ We recently reported an optimization of the DBU-CO_2_ switchable solvent system and could unambiguously proof that carbonate anions are indeed formed *in situ*.^22^ The formation of this *in situ* carbonate not only led to a mild solubilization of cellulose in DMSO, but also allowed an activation of the cellulose hydroxyl groups leading to a milder modification such as succinylation.^23^ In this regard, a high DS value of 2.6 was reported for reaction carried out at room temperature in 30 min.^23^ Tunge and co-workers reported a related approach of alcohol activation using CO_2_ in the absence of any base catalyst.^24^ In this case, allyl alcohols were reacted directly with CO_2_ leading to the formation of an *in situ* allyl-carbonate, which forms a π-allyl complex with a Pd-precursor that can then reacts with nucleophiles (here derived from nitroalkanes, nitriles and aldehydes) in a Tsuji–Trost like fashion.^24^

In the current contribution, the idea is to utilize this *in situ* generated carbonate anion as an acid component (*i.e.* nucleophile) in a typical P-3CR and is thus the starting point of the herein reported results. In this case, the CO_2_ is able to activate the alcohols and is incorporated as C1 carbon source into the desired compound. In this work, we thus report, for the first time, the Passerini four component reaction (P-4CR) as a variation of the P-3CR, achieved by replacing the acid component in the P-3CR by an alcohol and CO_2_. The utilization of CO_2_ as a carbon source is both interesting from an environmental and sustainable perspective as well as to extend the scope and achievable structural variety of MCRs.

## 2. Experimental

### Materials

Allyl-alcohol (99%), benzyl alcohol (99%), butanol (99.5%), 1-octanol (99%), phenyl acetaldehyde (98%, stabilized) and trimethylamine (99.7%) were purchased from Acros Organics. 1-Adamantyl isocyanide (95%), cyclohexanol (99%), cyclohexyl isocyanide (98%), 2-butanol (99.5%, anhydrous), 2,4-dinitrobenzaldehyde (97%), isobutyraldehyde (≥99.5%), 2-morpholinoethyl isocyanide (≥98%), *tert*-butanol (≥99.5%, anhydrous), *tert*-butyl isocyanide (98%), tetradecane (≥99.5%) and undecylenic aldehyde (95%) were obtained from Sigma Aldrich. Other chemicals used include: diazabicyclo[5.4.0]undec-7-ene (DBU, TCl, >98%), deuterated chloroform (CDCI_3_-*d*, Merck), dimethyl sulfoxide (DMSO, VWR, 99%) and trimethyl acetaldehyde (ABCR, 97%). Carbon dioxide (CO_2_) with purity over 99.9% was obtained from Air Liquide. Cyclohexane, ethyl acetate and dichloromethane were distilled before usage, while tetrahydrofuran (THF) was of technical grade and used without further purification. All other chemicals were used as received from the supplier.

### Instruments

#### IR spectroscopy

Infrared spectra of all samples were recorded on a Bruker alpha-p instrument using ATR technology within the range 4000 to 400 cm^−1^ with 24 scans.

#### Nuclear magnetic resonance spectroscopy (NMR)

^1^H NMR spectra were recorded on a 500 MHz WB Bruker Avance I spectrometer operating at a frequency of 499.97 MHz for ^1^H- and a frequency of 125.72 MHz for ^13^C-measurement on a 8 mm TXI probe head with actively shielded z-gradients (at *θ* = 0°) and on a 4 mm triple HCX MAS probe head (at *ca. θ* = 65°) at 298 K, regulated with a Bruker VTU-3000. Measurements were done at ambient temperature. Measurements were done in CDCl_3_ and data are reported in ppm relative to 7.26 ppm and 77.16 ppm for ^1^H and ^13^C, respectively.

#### Gas chromatography (GC-FID)

For GC measurements, a GC-2010 Plus instrument from Shimadzu with a polar column (Rxi-642Sil MS, length: 30 m, diameter: 0.25 mm, film thickness: 0.25 μm) and a flame-ionization detector (FID) was used. The sample (1 μL) was injected and vaporized at 250 °C. The column was heated from 50 to 280 °C at a rate of 10 K min^−1^.

#### Gas chromatography-mass spectrometry (GC-MS)

Electron impact (EI) analyses were conducted using a Varian 431-GC instrument with a capillary column Factor Four™ VF-5ms (30 m × 0.25 mm × 0.25 μm) and a Varian 210-MS ion trap mass detector. Scans were performed from 40 to 650 *m*/*z* at rate of 1 scan per second. The oven temperature program applied during the analysis was: initial temperature 95 °C, hold for 1 min, ramp at 15 °C min^−1^ to 200 °C, hold for 2 min., ramp at 15 °C min^−1^ to 300 °C, hold for 5 min. The injector transfer line temperature was set to 250 °C. Measurements were performed in the split–split mode (split ratio 50 : 1) using helium as carrier gas (flow rate 1.0 mL min^−1^).

#### Electron spray ionization-mass spectrometer (ESI-MS)

Spectra were recorded on a Q Exactive (Orbitrap) mass spectrometer (Thermo Fisher Scientific, San Jose, CA, USA) equipped with a HESI II probe to record high resolution electrospray ionization-MS (ESI-MS). Calibration was carried out in the *m*/*z* range 74–1.822 using premixed calibration solutions (Thermo Fisher Scientific). A constant spray voltage of 4.7 kV and a dimensionless sheath gas of 5 were employed. The S-lens RF level was set to 62.0, while the capillary temperature was set to 250 °C. All samples were dissolved at a concentration range of 0.05–0.01 mg mL^−1^ in a mixture of THF and MeOH (3 : 2) doped with 100 μmol sodium trifluoroacetate and injected with a flow of 5 μL min^−1^.

### General procedure for optimization study of P-4CR

0.41 g of Butanol (1 eq., 5.50 mmol) and 5 mol% tetradecane as internal GC standard (70 μL) were stirred in 1.5 mL of the solvent at room temperature for 1 to 2 minutes, after which a sample was collected for GC analysis. The mixture was then saturated with CO_2_ (5 bar) for 15 minutes. In the same manner, isobutyraldehyde was pre-saturated with CO_2_ (5 bar) for 10 minutes, after which both solutions were mixed and further saturated with 5 bar of CO_2_ for 10 to 15 minutes. Subsequently, *tert*-butylisocyanide was added and the reaction was performed under 10 bar of CO_2_ for 24 h at room temperature (22–24 °C). Samples were then collected over the course of the reaction and analyzed by gas chromatography (GC) in order to calculate the conversion and the relative percentage between the observed products (P-4CR, P-3CR and hydrolysis product of P-4CR). Parameters investigated during the optimization study include: effect of reaction equivalents (1, 2 eq.), reaction concentration (1.84 and 3.68 M with respect to butanol), catalyst (triethylamine and DBU), catalyst concentration (5, 10 and 15 mol%), CO_2_ pressure (5, 10 and 15 bar) and solvent (DMSO, chloroform, methyl-THF, DCM).

## 3. Results and discussions

**Scheme 1:**
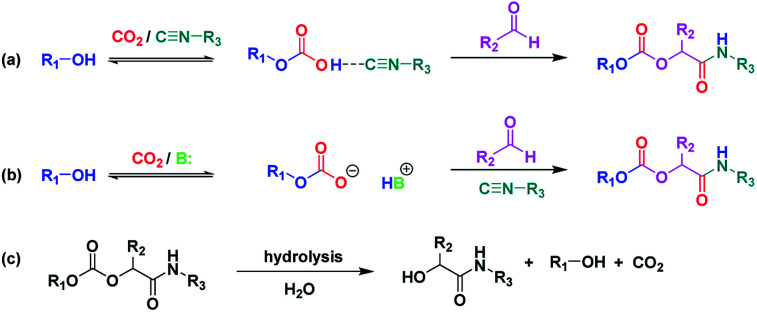
Formation of P-4CR products in the absence (a) and presence (b) of a base catalyst. (c) Hydrolysis of the P-4CR product. (see Fig. 2 for synthesized structures).

**Fig. 1:**
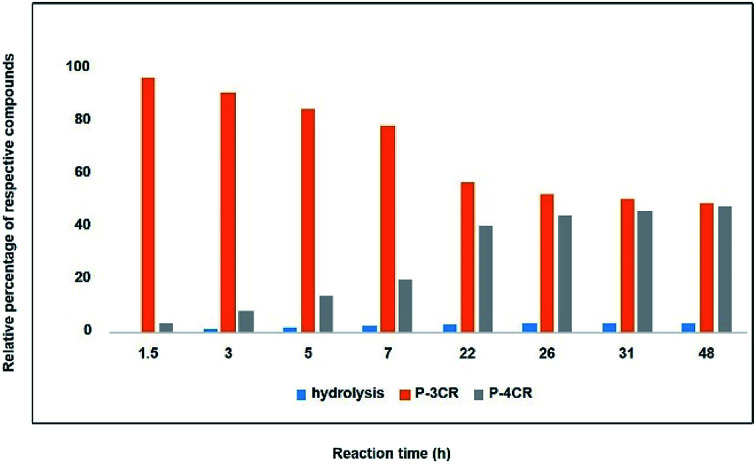
Relative percentage between formed compounds (P-4CR, P-3CR and hydrolysis of P-4CR products, compare Scheme 1) over time (results obtained from GC). Reaction conditions: One equivalent each of butanol, isobutyraldehyde and *tert*-butylisocyanide at 10 bar CO_2_ in DCM at room temperature.

As a starting point and to prove our hypothesis of a possible P-4CR, we investigated the reaction of butanol, isobutyraldehyde, *tert*-butylisocyanide and CO_2_ in dichloromethane (DCM) as a solvent. In addition to the formation of the expected P-4CR product, the formation of a P-3CR by-product and the hydrolysis of the P-4CR product, resulting in the formation of an α-hydroxyl-amide, was observed (compare Scheme 1). As we observed in the further course of our investigations, the formation of the P-3CR product is due to the presence of the respective carboxylic acids originating from the oxidation of the used aldehyde component. The isobutyraldehyde used for the optimizations had a lower purity (92% from ^1^H NMR) than reported by the manufacturer (99.5%). We later observed that freshly distilled aldehydes gave the best results for a P-4CR and the P-3CR could be suppressed to less than 5%, although it could not be completely avoided. Nevertheless, this first proof-of-principle reaction clearly showed that the anticipated reactivity of the carbonate anion allows for a P-4CR. For optimizing the P-4CR, several parameters, such as the effect of solvent, reactant concentration, reactant equivalents, catalyst concentration and CO_2_ pressure were investigated. Dichloromethane (DCM, 1.84 M) was employed as solvent for the first trial experiments as it has been reported to be a suitable solvent for the P-3CR.^25^ One equivalent of butanol, isobutyraldehyde and *tert*-butylisocyanide were employed, while the reaction was performed at 10 bar CO_2_ for 48 h at room temperature. The conversion of butanol was followed *via* gas chromatography (GC) *versus* an internal standard (tetradecane). For the first trial, a conversion of 38% was reached after 48 h (see Fig. SI1†). The presence of the desired P-4CR product (273.19 g mol^−1^) was indicated by gas chromatography mass spectrometry (GC-MS). In addition, the presence of the hydrolysis product of the P-4CR (174.10 g mol^−1^) as well as the P-3CR product (243.16 g mol^−1^) were confirmed by GC-MS. The formation of the three products was followed in time *via* GC and is illustrated in Fig. 1. Within the first five hours, the P-3CR accounted for most of the observed products. However, an increased formation of the P-4CR was observed as the reaction proceeded. In addition, some hydrolysis of the P-4CR product was observed with time. Considering the P-3CR side reaction, a two-fold excess of aldehyde and isocyanide components was employed in the next reaction, resulting in an improved butanol conversion of 56%.

As solvents play a key role in multicomponent reactions, different solvents (dimethyl sulfoxide DMSO, methyl-THF and chloroform) were investigated next. While similar conversions were obtained for all the solvents investigated, different hydrolysis tendencies were noticed. By comparing the relative percentage between the P-4CR and its hydrolysis product, an increased formation of hydrolysis product in the order chloroform > DMSO > methyl-THF > DCM was observed. This trend might be explained considering the acidity of the solvents, *e.g.* chloroform is the most acidic solvent tested herein. For DMSO, the high hydrolysis rate observed is probably due to the presence of unavoidable water impurity in the solvent. Methyl-THF showed a relatively low tendency towards hydrolysis, but led to a higher degree of oxidation of the aldehyde and thus an increase of the P-3CR side product (see Fig. SI2†), probably due to possible peroxide impurities.^26^ DCM showed the lowest tendency towards hydrolysis and equally resulted in the highest selectivity towards the targeted P-4CR product. It should however be noted here that the observed hydrolysis products are valuable compounds as well. Furthermore, as multicomponent reactions usually provide higher efficiency at higher concentrations, this parameter was investigated by doubling the previously applied concentration. As expected and to our delight, the conversion of butanol increased from 56% (1.84 M with respect to butanol) to 73% (3.68 M with respect to butanol) in DCM.

Presumably, the mechanism of the P-4CR proceeds *via* the *in situ* formation of a carbonate anion initiated by a base catalyst^16,20–22^ (see Scheme 1b). The carbonate then reacts as the acid component with the isocyanide and aldehyde component in the typical fashion of the conventional Passerini 3-CR.^3^ However, in the so far discussed set of experiments, no base catalyst was employed. It was therefore interesting to note that the P-4CR occurred nevertheless. This initially unexpected reactivity could be due to an activation of the alcohol by the isocyanide (Scheme 1a). Isocyanides were used as a weak Lewis base in previous reports in literature.^27^ Equally, alcohol activation by other Lewis bases has been reported.^28^ To verify the alcohol activation hypothesis, an *in situ*^1^H NMR study was performed (utilizing butanol in CDCl_3_). In this context, the spectra of butanol, butanol in the presence of stoichiometric amounts of *tert*-butylisocyanide and butanol in the presence of stoichiometric amounts of Et_3_N were compared. The respective study is displayed in the ESI (Fig. SI3†) and is described below.

From this NMR study, a slight downfield chemical shift of the OH proton (from 3.16 ppm to 3.20 ppm) and significant signal broadening, characteristic of H-bonding, was observed for butanol in the presence of *tert*-butylisocyanide compared to the spectrum of butanol without isocyanide in CDCl_3_. Similar proton signal broadening of the OH proton, most probably caused by H-bonding, has also been reported for Schiff bases.^29^ Comparing the spectrum of butanol with the spectrum of butanol in the presence of Et_3_N, a downfield chemical shift of the OH proton (from 3.16 ppm to 4.38 ppm) was observed, while displaying similar signal broadening (see Fig. SI3b†). These results indicate H-bonding of the OH proton and hence, activation of the alcohol by the isocyanide acting as a Lewis base.

The effect of a catalyst was thus evaluated next. As mentioned above, a basic catalyst is able to activate the hydroxyl group, thereby increasing the carbonate anion formation. Thus, triethylamine (10 mol%) was tested. The obtained results were compared with the reaction without addition of the catalyst (see Fig. SI4a†). As expected, the presence of the catalyst further accelerated the reaction. A conversion of almost 70% was reached within 24 h (compared to 48 h required to achieve similar conversion in the absence of the catalyst). In addition, the overall selectivity towards P-4CR increased (see Fig. SI4b and c†). However, a slight increase in hydrolysis product was also observed in the presence of the base catalyst, as one could expect (see Fig. SI4b and c†). In another set of experiments, the two catalysts Et_3_N and diazabicyclo[5.4.0]undec-7-ene (DBU) were compared. The obtained results showed similar conversion of butanol (about 70%) after 24 h. However, an increased hydrolysis was observed for the reaction with DBU compared to Et_3_N (Fig. SI5†), probably due to the higher basicity of DBU compared to Et_3_N. In addition, the formation of the P-3CR side product was less pronounced for Et_3_N. Hence, Et_3_N was used for the following experiments, screening for catalyst concentration employing 5, 10 and 15 mol%. This study revealed that the conversion of butanol remained at about 70% for both 5 and 10 mol%. However, the quasi conversion decreased to 52% when 15 mol% catalyst were used. This decrease can be explained by the observed increased hydrolysis of the P-4CR product, which reforms butanol and thus counterfeits a lower butanol conversion (see Scheme 1c and Fig. SI6†). As 10 mol% catalyst loading gave slightly better results, this catalyst loading was employed for investigating the effect of CO_2_ pressure. Three CO_2_ pressures were investigated (5, 10 and 15 bar) and the results are presented in Fig. SI7,† revealing a slight increase in butanol conversion from 58% to 69% as the CO_2_ pressure was increased from 5 to 10 bar. However, a slight decrease was observed at 15 bar (62% conversion). Furthermore, the highest relative percentage of P-4CR was obtained at 10 bar of CO_2_. Therefore, 10 bar of CO_2_ was selected for further experiments.

**Fig. 2:**
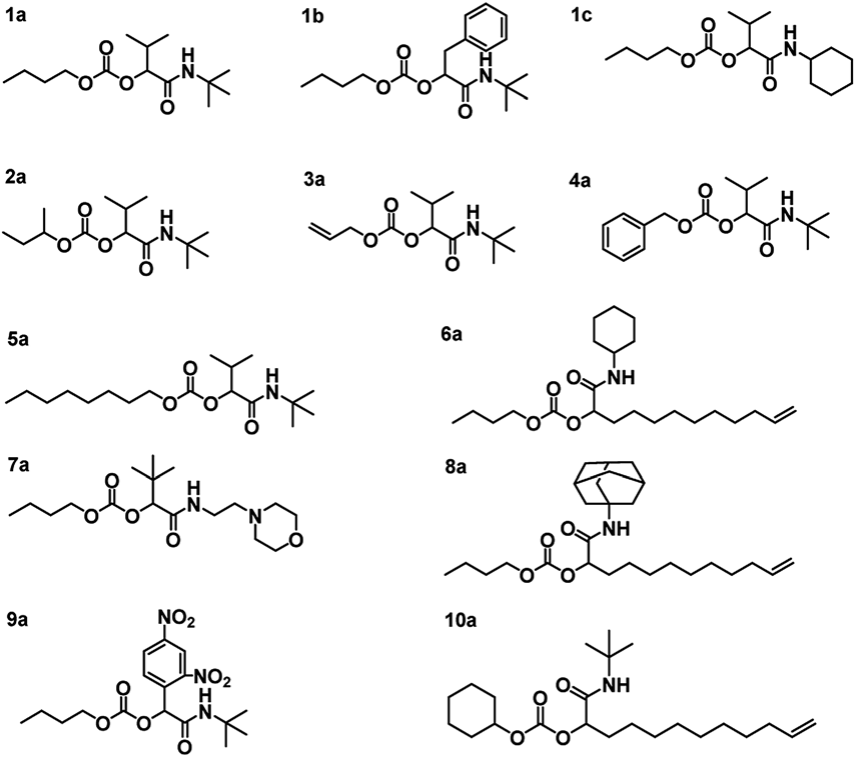
Structures of the synthesized P-4CR products.

**Fig. 3:**
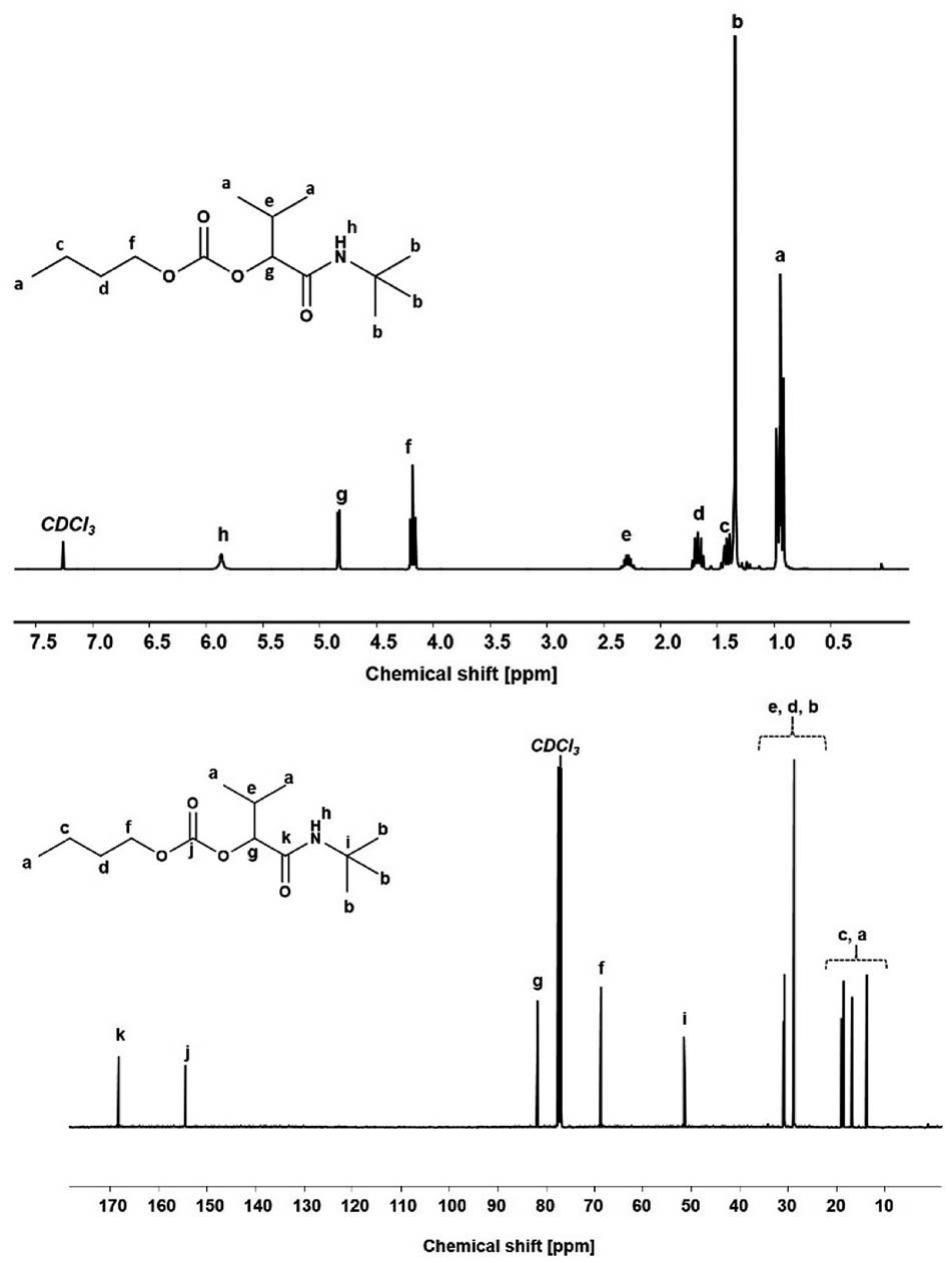
^1^H NMR (top) and ^13^C NMR (bottom) of P-4CR product 1a.

Using the described optimized conditions (3.84 M with respect to butanol, DCM as solvent, 1 eq. of alcohol, 2 eq. of aldehyde and isocyanide with respect to butanol, 10 bar CO_2_ at room temperature (22–25 °C)), the scope of the P-4CR was investigated by varying the used components (see Fig. 2). For the synthesis of 1a, butanol, isobutyraldehyde and *tert*-butylisocyanide were used. A conversion of about 70% was reached within 24 h, longer reaction times (up to 45 h) led to an increase of selectivity of the targeted P-4CR compound 1a. Compound 1a was isolated in 43% yield after column chromatography. This value is within the range of previously reported Ugi 5-CR products.^6,7^ Keating *et al.* reported similar results when other alcohols apart from methanol were used in this Ugi-5CR.^6^ It is also important to point out that in the case of methanol, an excess (over 10 times) was utilized in order to achieve high conversion and yields. The structure of 1a was confirmed *via*^1^H and ^13^C NMR performed in CDCl_3_ as shown in Fig. 3.

The attribution of the proton and carbon peaks are similar to previous reports on the P-3CR,^25^ as well as Ugi-5CR.^7^ In the ^1^H NMR spectrum, the amide proton is visible at 5.87 ppm. The proton signal at the tertiary carbon at 4.83 ppm and the 9H from the *tert*-butyl side chain at 1.35 ppm confirm the proposed structure. From the ^13^C NMR, two characteristic carbonyl carbon chemical shifts are observed at 168.78 ppm (amide) and 154.93 ppm (carbonate). Furthermore, the tertiary carbon in the isocyanide side chain is observed at 51.70 ppm. In addition, from the FT-IR spectrum (see Fig. SI8†), the characteristic C=O stretching absorbance band of the carbonate (1743 cm^−1^) and amide (1654 cm^−1^) were visible. Finally, the mass of the molecule was confirmed by ESI-MS ([C_14_H_27_NO_4_Na]^+^ = 296.19 g mol^−1^, obtained = 296.18 g mol^−1^).

Employing similar conditions, a total of twelve molecules were synthesized and equally characterized (see Fig. 2). On the example of butanol, five variations of the aldehyde and four isocyanide variations were demonstrated. In addition, a secondary alcohol (2-butanol), an unsaturated alcohol (allyl-alcohol), benzyl-alcohol, octanol and cyclohexanol were utilized. The respective P-4CR products were obtained with conversions ranging from 16 to 82% and isolated yields between 18 and 43%. Lower conversions were obtained for reactions involving sterically demanding side chains such as 1-adamantyl-7a (19%) and 2-morpholinoethyl-8a (16%). In the course of these investigations, we observed that the P3-CR side-reaction can be suppressed to less than 5% by immediate utilization of a freshly distilled aldehyde component.

The obtained P-4CR compounds were fully characterized *via*^1^H and ^13^C NMR (see ESI†), FT-IR and high-resolution mass spectrometry (ESI-MS). In the case of 1b, employing phenyl acetaldehyde, the P-3CR side reaction was not observed. However, in this case (phenyl acetaldehyde), the presence of a proton in α-position to the aromatic ring led to an aldol condensation side-product. The aldol side reaction was favoured when the base catalyst was used, as expected. Hence, for this reaction, no catalyst was utilized and a butanol conversion of 36% was reached after 24 h (without much improvement with longer reaction time). Noteworthy, the P-4CR products from benzyl-alcohol 4a and octanol 5a were less prone to hydrolysis. Their higher hydrophobicity compared to butanol might explain this tendency. The reaction performed with allyl alcohol 3a required longer reaction times to achieve suitable conversions (30 h, conversion 50%) and showed the highest tendency towards hydrolysis, which also increased over time. Running the reaction even longer (48 h) resulted in 82% conversion. The P-4CR product was isolated in a yield of 33%, while 23% of the hydrolysis product were isolated (and characterized *via* the same techniques). This result confirms that hydrolysis can also be used on purpose to obtain equally useful α-hydroxyl-amide products. Our attempt to introduce an aromatic side chain *via* the aldehyde component (benzaldehyde) was unsuccessful, as only the P-3CR and hydrolysis products were formed. In addition, benzaldehyde is very easily oxidized to the corresponding acid (despite distillation before usage). Thus, 2,4-dinitrobenzaldehyde was used instead to obtain 9a. A conversion of 40% was achieved after 48 h with 22% isolated yield. In a similar manner, we could not observe the formation of the P-4CR product when *tert*-butanol was used. Again, only P-3CR and the hydrolysis product of P-4CR were observed *via* GC-MS. Nevertheless, this demonstrate that also tertiary alcohols can be used in this reaction, whereby only one of the two possible products (*i.e.* the hydrolysis product) is accessible so far. Finally, the hydrolysis products of 1c, 3a and 9a could be isolated in a yield of 30%, 23% and 63%, respectively. Their structures were confirmed *via*^1^H and ^13^C NMR (see ESI†).

## 4. Conclusion

We report a novel variant of the Passerini reaction, the Passerini four component reaction (P-4CR), by replacing the carboxylic acid component in a conventional P-3CR with an alcohol and CO_2_. Upon optimization of the reaction parameters (reaction time, reactant equivalents, reactant concentration, solvent, catalyst concentration and CO_2_ pressure), twelve P-4CR products were successfully synthesized with conversions ranging from 16 to 82% and isolated yields between 18 and 43%. In addition, hydrolysis of the P-4CR products, leading to the formation of α-hydroxyl-amides, was observed. Three of these hydrolysis products were isolated with yields between 23 and 63%. Furthermore, the formation of P-3CR products was observed, which occurred due to the oxidation of the employed aldehyde components. The success of our reported P-4CR does not only expand the structural diversity of multicomponent reactions, but the direct utilization and activation of CO_2_ as a C1 building block is equally noteworthy.

## Conflicts of interest

The authors declare no conflict of interest.

## Supplementary Material

RA-008-C8RA07150K-s001
