# Supplementary material for: On the direct use of CO2 in multicomponent reactions: introducing the Passerini four component reaction
Source: RSC Adv. 2018 Sep 7;8(55):31490–5. doi: 10.1039/c8ra07150k (PMC9085615; doi:10.1039/c8ra07150k)
Supplement: RA-008-C8RA07150K-s001 [file RA-008-C8RA07150K-s001.pdf]

Electronic Supplementary Material (ESI) for RSC Advances.
This journal is © The Royal Society of Chemistry 2018

Supporting Information for:

On the direct use of CO_2 in multicomponent reactions: Introducing the Passerini four component reaction

Kelechukwu N. Onwukamike,[a,b,c] *Stéphane Grelier,*[b,c] *Etienne Grau,*[b,c] *Henri Cramail,*[b,c*] *Michael A.R. Meier*[a*]

[a] Institute of Organic Chemistry (IOC), Materialwissenschaftliches Zentrum (MZE), Karlsruhe Institute of Technology (KIT), Straße am Forum 7, 76131 Karlsruhe, Germany

[b] Univ. Bordeaux, CNRS, Bordeaux INP/ENSCBP, Laboratoire de Chimie des Polymères Organiques, UMR 5629, 16 avenue Pey-Berland, F-33607 Pessac Cedex, France

[c] Centre National de la Recherche Scientifique, Laboratoire de Chimie des Polymères Organiques, UMR 5629, 16 avenue Pey-Berland, F-33607 Pessac Cedex, France

I.	EXPERIMENTAL SECTION	S2
II.	OPTIMIZATION STUDIES OF THE P-4CR	S3
III.	FT-IR CHARACTERIZATION OF P-4CR PRODUCT **1a**	S7
IV.	SYNTHESIS AND CHARACTERIZATION OF P-4CR PRODUCTS	S8
V.	CHARACTERIZATION OF HYDROLYSIS PRODUCTS OF P-4CR	S31

I. EXPERIMENTAL SECTION

Materials

Allyl-alcohol (99%), benzyl alcohol (99%), butanol (99.5%), 1-octanol (99%), phenyl acetaldehyde (98%, stabilized) and trimethylamine (99.7%) were purchased from Acros Organics. 1-adamantyl isocyanide (95%), cyclohexanol (99%), cyclohexyl isocyanide (98%), 2-butanol (99.5%, anhydrous), 2,4-dinitrobenzaldehyde (97%), isobutyraldehyde (≥ 99.5%), 2-morpholinoethyl isocyanide (≥ 98%), *tert*-butanol (≥ 99.5%, anhydrous), *tert*-butyl isocyanide (98%), tetradecane (≥ 99.5%) and undecylenic aldehyde (95%) were obtained from Sigma Aldrich. Other chemicals used include: diazabicyclo[5.4.0]undec-7-ene (DBU, TCl, > 98%), deuterated chloroform ($CDCl_3$-*d,* Merck), dimethyl sulfoxide (DMSO, VWR, 99%) and trimethyl acetaldehyde (ABCR, 97%). Carbon dioxide (CO_2) with purity over 99.9% was obtained from Air Liquide. Cyclohexane, ethylacetate and dichloromethane were distilled before usage, while tetrahydrofuran (THF) was of technical grade and used without further purification. All other chemicals were used as received from the supplier.

Instruments

IR spectroscopy. Infrared spectra of all samples were recorded on a Bruker alpha-p instrument using ATR technology within the range 4000 to 400 cm^{-1} with 24 scans.

Nuclear Magnetic Resonance Spectroscopy (NMR). ^{1}H NMR spectra were recorded on a 500 MHz WB Bruker Avance I spectrometer operating at a frequency of 499.97 MHz for ^{1}H- and a frequency of 125.72 MHz for ^{13}C-measurement on a 8 mm TXI probe head with actively shielded z-gradients (at θ =0°) and on a 4 mm triple HCX MAS probe head (at ca. θ = 65°) at 298 K, regulated with a Bruker VTU-3000. Measurements were done at ambient temperature. Measurements were done in $CDCl_3$ and data are reported in ppm relative to 7.26 ppm and 77.16 ppm for ^{1}H and ^{13}C, respectively.

Gas chromatography (GC-FID). For GC measurements, a GC-2010 Plus instrument from Shimadzu with a polar column (Rxi-642Sil MS, length: 30 m, diameter: 0.25 mm, film thickness: 0.25 µm) and a flame-ionization detector (FID) was used. The sample (1 µL) was injected and vaporized at 250 °C. The column was heated from 50 to 280 °C at a rate of 10 $Kmin^{-1}$.

*Gas chromatography-Mass spectrometry (GC-MS)***.** Electron impact (EI) analyses were conducted using a Varian 431-GC instrument with a capillary column Factor Four™ VF-5ms

(30 m × 0.25 mm × 0.25 µm) and a Varian 210-MS ion trap mass detector. Scans were performed from 40 to 650 m/z at rate of 1 scan per second. The oven temperature program applied during the analysis was: initial temperature 95 °C, hold for 1 min, ramp at 15 °C·min^{-1} to 200 °C, hold for 2 min., ramp at 15 °C·min^{-1} to 300 °C, hold for 5 min. The injector transfer line temperature was set to 250 °C. Measurements were performed in the split-split mode (split ratio 50:1) using helium as carrier gas (flow rate 1.0 mL min^{-1}).

*Electron Spray Ionization-Mass Spectrometer (ESI-MS)***.** Spectra were recorded on a Q Exactive (Orbitrap) mass spectrometer (Thermo Fisher Scientific, San Jose, CA, USA) equipped with a HESI II probe to record high resolution electrospray ionization–MS (ESI-MS). Calibration was carried out in the *m/z* range 74–1.822 using premixed calibration solutions (Thermo Fisher Scientific). A constant spray voltage of 4.7 kV and a dimensionless sheath gas of 5 were employed. The S-lens RF level was set to 62.0, while the capillary temperature was set to 250 °C. All samples were dissolved at a concentration range of 0.05 – 0.01 mgmL^{-1} in a mixture of THF and MeOH (3:2) doped with 100 µmol sodium trifluoroacetate and injected with a flow of 5 µLmin^{-1}.

II. OPTIMIZATION STUDY OF THE P-4-CR

General procedure for optimization study of P-4CR

0.41 g of Butanol (1 eq., 5.50 mmol) and 5 mol% tetradecane as internal GC standard (70 µL) were stirred in 1.5 mL of the solvent at room temperature for 1 to 2 minutes, after which a sample was collected for GC analysis. The mixture was then saturated with CO_2 (5 bar) for 15 minutes. In the same manner, isobutyraldehyde was pre-saturated with CO_2 (5 bar) for 10 minutes, after which both solutions were mixed and further saturated with 5 bar of CO_2 for 10 to 15 minutes. Subsequently, *tert*-butylisocyanide was added and the reaction was performed under 10 bar of CO_2 for 24 h at room temperature (22-24 °C). Samples were then collected over the course of the reaction and analyzed by Gas chromatography (GC) in order to calculate the conversion and the relative percentage between the observed products (P-4CR, P-3CR and hydrolysis product of P-4CR). Parameters investigated during the optimization study include: effect of reaction equivalents (1, 2 eq.), reaction concentration (1.84 and 3.68 M with respect to butanol), catalyst (triethylamine and DBU), catalyst

concentration (5, 10 and 15 mol%), CO_2 pressure (5, 10 and 15 bar) and solvent (DMSO, chloroform, methyl-THF, DCM).

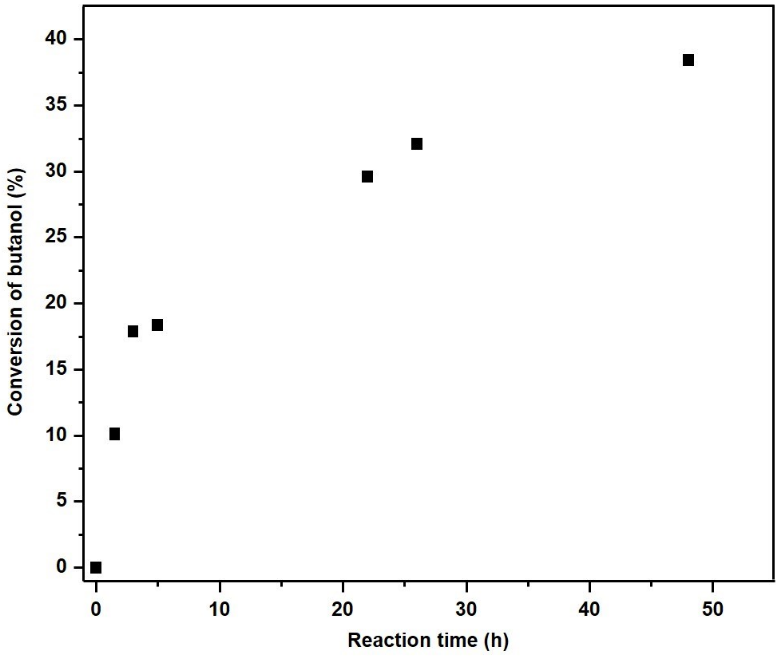

Figure SI 1*:* Conversion of butanol over time observed via GC-MS (1.84 M, 1 eq. isobutyraldehyde, 1 eq. *tert*-butylisocyanide, 10 bar CO_2, 48 h at room temperature)

Effect of solvent on the relative ratio between observed products

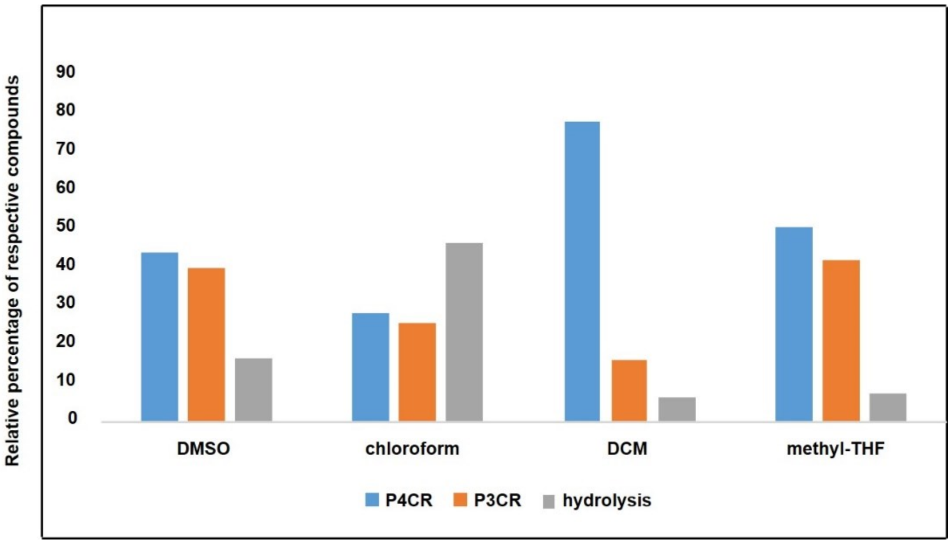

Figure SI 2*:* Effect of solvent on the relative percentage between P-4CR, P-3CR and hydrolysis products (1.84 M, 2 eq. isobutyraldehyde, 2 eq. *tert*-butylisocyanide, 10 bar CO_2, 24 h at room temperature). Values obtained through GC measurement with tetradecane as internal standard.

^{1}H NMR study for verifying the Lewis basic behaviour of isocyanide

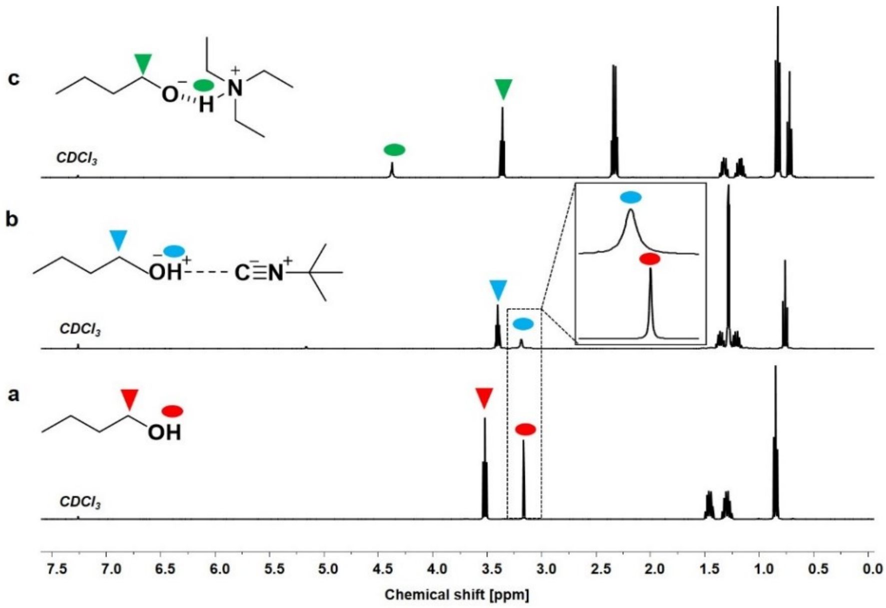

Figure SI 3: ^{1}H ($CDCl_3$) NMR comparison between butanol (**a**), butanol and *tert*-butylisocyanide (**b**), butanol and Et_3N (**c**).

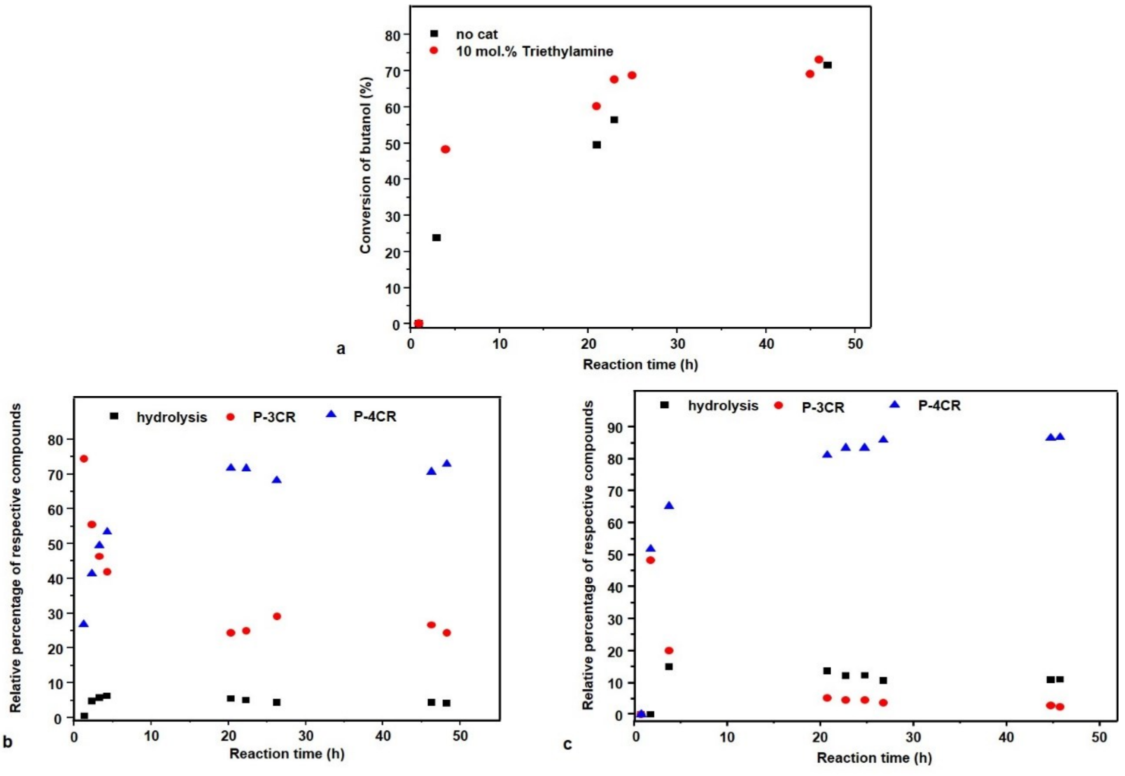

***Figure SI 4**:* Results obtained by GC of: **a**. Influence of catalyst (10 mol. % Et_3N) on the conversion of butanol over time compared to the reaction without any catalyst (3.68 M in DCM, 2 eq. isobutyraldehyde, 2 eq. *tert*-butylisocyanide, 10 bar CO_2, 45-48 h at room temperature). **b**. Relative percentage of respective products (P-4CR, P-3CR and hydrolysis of P-4CR) for the reaction performed without catalyst. **c**. Relative percentage of respective products (P-4CR, P-3CR and hydrolysis of P-4CR) for the reaction performed with 10 mol% Et_3N as catalyst. (**b** and **c;** values are obtained after normalization using tetradecane).

Catalyst comparison on the relative percentage between observed products

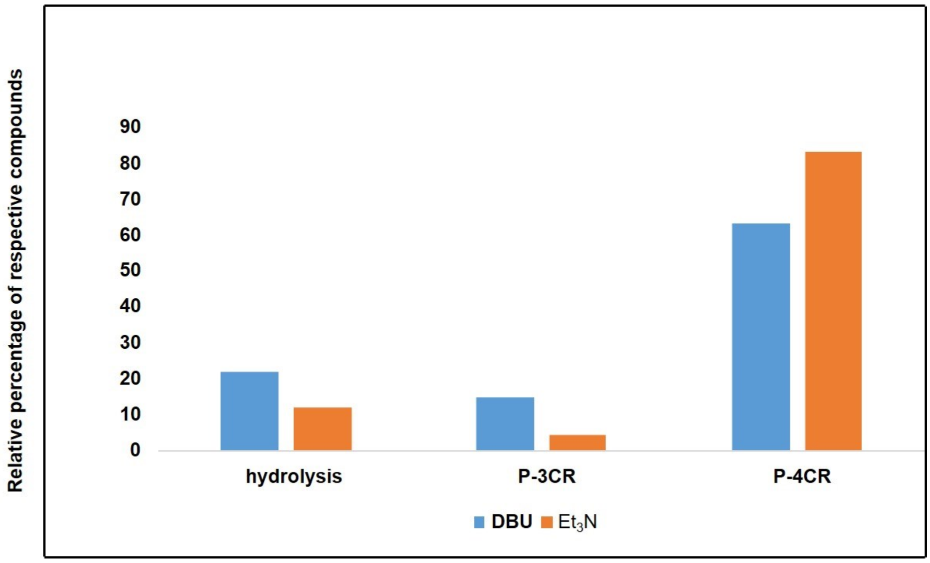

***Figure SI 5**:* Comparison between triethylamine and DBU as catalyst on the relative percentage between P-4CR, P-3CR and hydrolysis products (1.84 M, 2 eq. isobutyraldehyde, 2 eq. *tert*-butylisocyanide, 10 bar CO_2, 24 h at room temperature). Values obtained through GC measurement with tetradecane as internal standard.

Effect of triethylamine catalyst concentration on the relative ratio between observed products

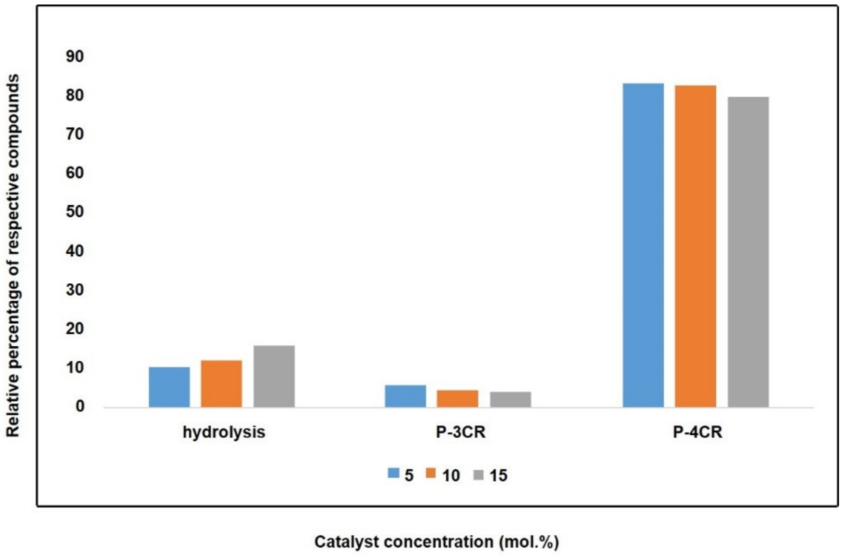

***Figure SI 6**:* Effect of catalyst concentration using triethylamine on the relative percentage between P-4CR, P-3CR and hydrolysis products (1.84 M, 2 eq. isobutyraldehyde, 2 eq. *tert*-butylisocyanide, 10 bar CO_2, 24 h at room temperature). Values obtained through GC measurement with tetradecane as internal standard.

Effect of CO_2 pressure on the relative ratio between observed products

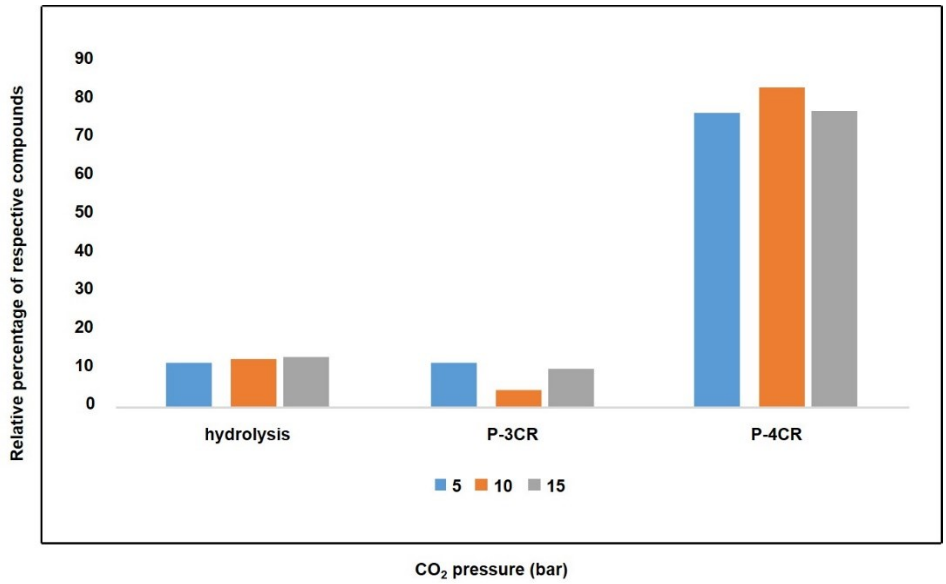

Figure SI 7*:* Effect of CO_2 pressure on the relative percentage between P-4CR, P-3CR and hydrolysis products (10 mol.% triethylamine, 1.84 M, 2 eq. isobutyraldehyde, 2 eq. *tert*-butylisocyanide, 10 bar CO_2, 24 h at room temperature). Values obtained through GC measurement with tetradecane as internal standard.

III. FT-IR CHARACTERIZATION OF P-4CR PRODUCT 1a

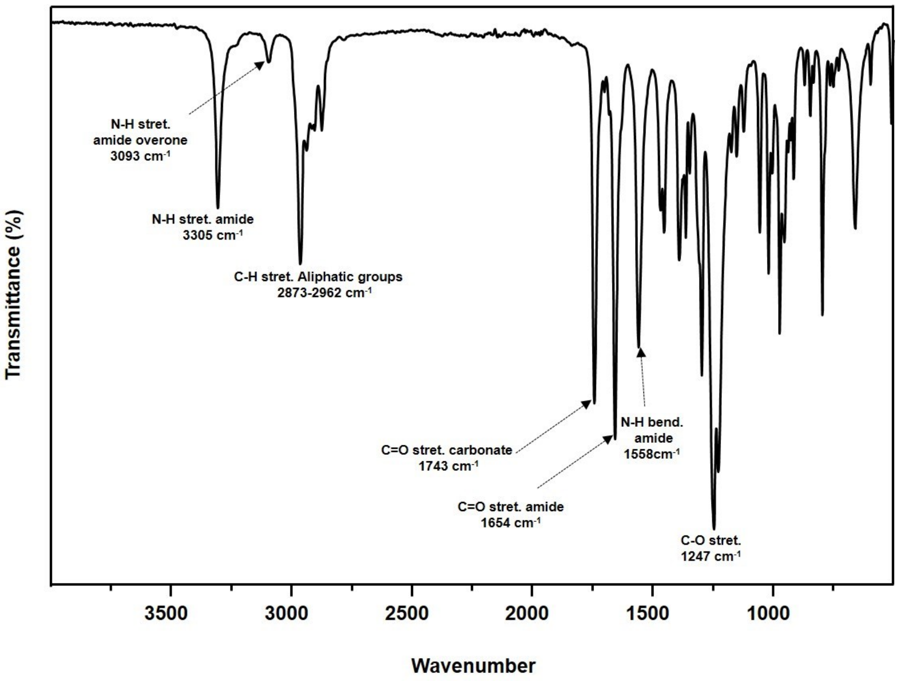

Figure SI 8*:* FT-IR spectrum of P-4CR product **1a**.

IV. SYNTHESIS OF P-4CR PRODUCTS

Molecule 1a. Butyl (1-(*tert*-butylamino)-3-methyl-1-oxobutan-2-yl) carbonate

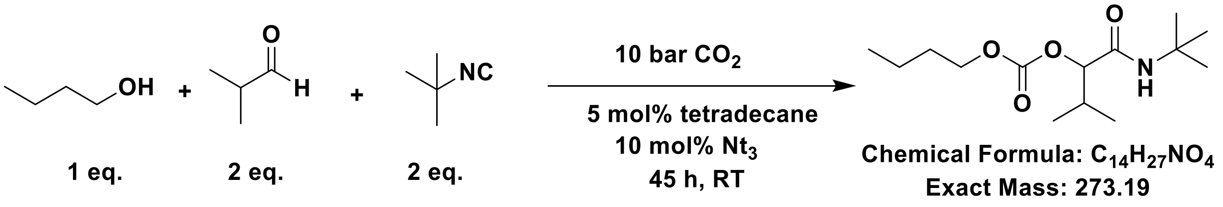

0.41 g of butanol (1 eq., 5.50 mmol) and 5 mol% tetradecane standard (70 µL) were stirred in 1.5 mL of dichloromethane (DCM) at room temperature for 1 to 2 minutes, after which a sample was collected for GC analysis (T_0). 10 mol% triethylamine catalyst (80 µL) was added and the mixture was saturated with CO_2 (5 bar) for 15 minutes. In the same manner, 0.79 g of isobutyraldehyde (2 eq., 11 mmol, 1.0 mL) was pre-saturated with CO_2 (5 bar) for 10 minutes, after which both solutions were mixed and further saturated with 5 bar of CO_2 between 10-15 minutes. Furthermore, 0.91 g of *tert*-butyl isocyanide (2 eq., 11 mmol, 1.24 mL) was added and the reaction performed under 10 bar of CO_2 for 45 h at room temperature (22-24 °C). After 45 h, a sample was collected for GC analysis (T_f) in order to calculate the conversion and relative ratio values between observed products. The crude mixture was concentrated via rotary evaporation and purified via column chromatography (gradient CH:EE starting from 15:1 to 5:1). The product was obtained as white powder. Conversion: 73%, isolated yield: 43%.

^{1}H NMR (500 MHz, $CDCl_3$) δ (ppm) = 5.87 (s, NH, 1H), 4.83 (d, CH, 1H), 4.18 (t, CH_2, 2H), 2.29 (m, CH, 1H), 1.67 (dt, CH_2, 2H), 1.41 (dt, CH_2, 2H), 1.35 (s, 3 CH_3, 9H), 0.94 (dt, 3 CH_3, 9H).

^{13}C NMR (125 MHz, $CDCl_3$) δ (ppm) = 168.76 (k), 154.93 (j), 82.01 (g), 69.03 (f), 51.70 (i), 31.11 (e), 30.97 (d), 29.06 (b), 19.26 (c), 18.93, 16.97, 14.03 (a).

ATR-IR: ν (cm^{-1}) = 3305.1, 3093.4, 2962.3, 2873.8, 1743.6, 1654.3, 1558.1, 1451.0, 1388.3, 1246.8, 970.3, 799.8, 660.3, 509.0.

Exact mass: $[C_{14}H_{27}NO_4Na]^+$ = 296.19 $gmol^{-1}$, obtained (ESI-MS) = 296.18 $gmol^{-1}$

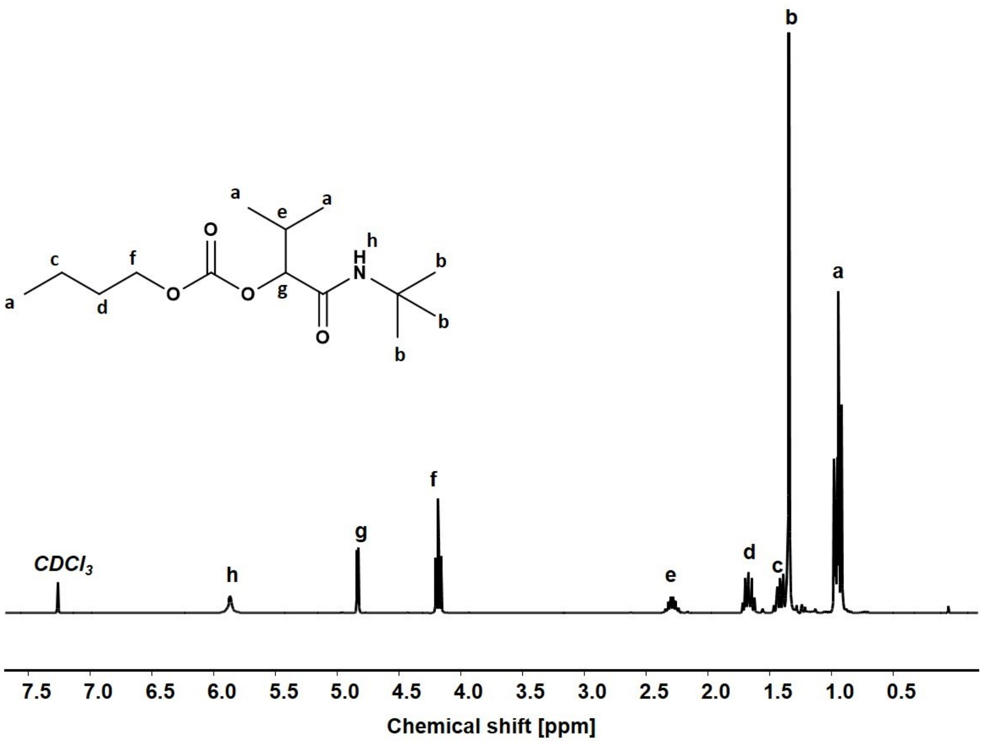

Figure SI 9a: ^{1}H ($CDCl_3$) NMR of P-4CR **1a.**

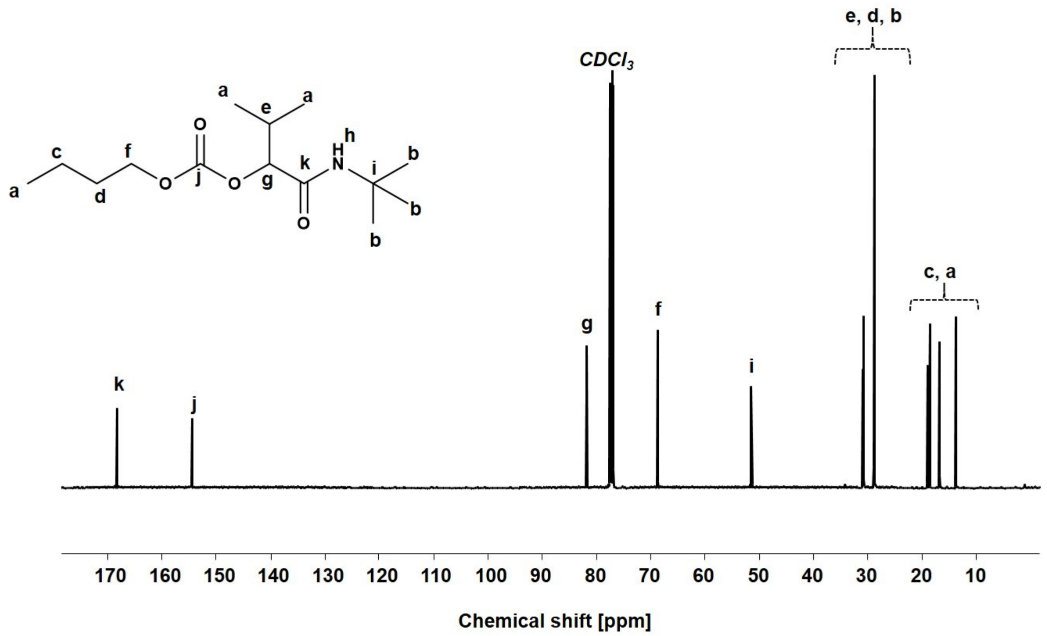

Figure SI 9b: ^{13}C ($CDCl_3$) NMR of P-4CR **1a.**

Molecule 1b. Butyl (1-(*tert*-butylamino)-1-oxo-3-phenylpropan-2-yl) carbonate

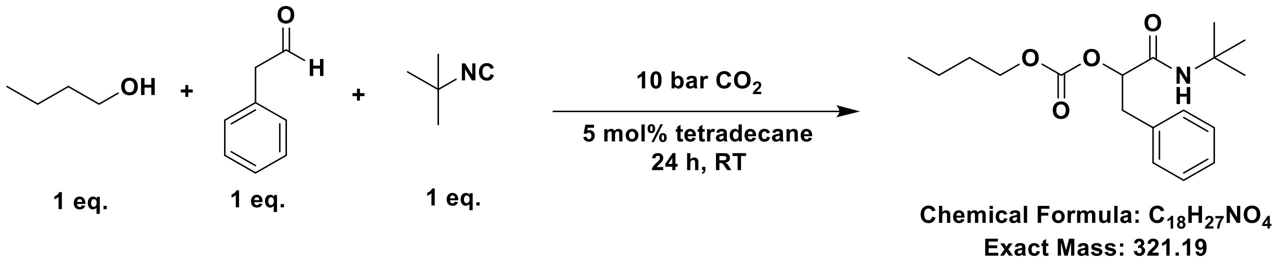

0.41 g of Butanol (1 eq., 5.50 mmol) and 5 mol% tetradecane standard (70 µL) were stirred in 1.5 mL of dichloromethane (DCM) at room temperature for few minutes, after which a sample was collected for GC analysis (T_0). Next, the mixture was saturated with CO_2 (5 bar) for 15 minutes. In a similar fashion, 0.66 g of phenyl acetaldehyde (1 eq., 5.5 mmol, 0.62 mL) was pre-saturated with CO_2 (5 bar) for 10 minutes, after which both solutions were mixed and further saturated with 5 bar of CO_2 between 10-15 minutes. Furthermore, 0.46 g of *tert*-butyl isocyanide (1 eq., 5.5 mmol, 0.62 mL) was added and the reaction was performed under 10 bar of CO_2 for 24 h at room temperature (22-24 °C). After 24 h, a sample was collected for GC analysis (T_f) in order to calculate the conversion and relative ratio values between the observed products. The crude mixture was concentrated via rotary evaporation and purified *via* column chromatography (gradient CH:EE starting from 20:1 to 5:1). The product was obtained as white powder. Conversion: 36%, isolated yield: 25%.

^{1}H NMR (500 MHz, $CDCl_3$) δ (ppm) = 7.26-7.18 (td, Ar-H, 5H), 5.66 (s, NH, 1H), 5.08 (dd, CH, 1H), 4.05 (m, CH_2, 2H), 3.09 (m, CH_2, 2H), 1.55 (m, CH_2, 2H), 1.30 (dq, CH_2, 2H), 1.19 (s, 3 CH_3, 9H), 0.87 (t, 3 CH_3, 9H).

^{13}C NMR (125 MHz, $CDCl_3$) δ (ppm) = 167.85 (m), 154.05 (l), 135.72 (k), 130.12 128.26, 127.03 (i), 77.68 (g), 68.77 (f), 51.45 (i), 37.94 (e) 30.66 (d), 28.89 (b), 19.11 (c), 13.76 (a).

ATR-IR: ν (cm^{-1}) = 3297.7, 3085.6, 3032.7, 2963.6, 2933.0, 2872.5, 1755.2, 1659.9, 1554.9, 1246.3, 1125.6, 699.2, 511.3.

Exact mass: $[C_{18}H_{27}NO_4Na]^+$ = 344.19 $gmol^{-1}$, obtained (ESI-MS) = 344.18 $gmol^{-1}$

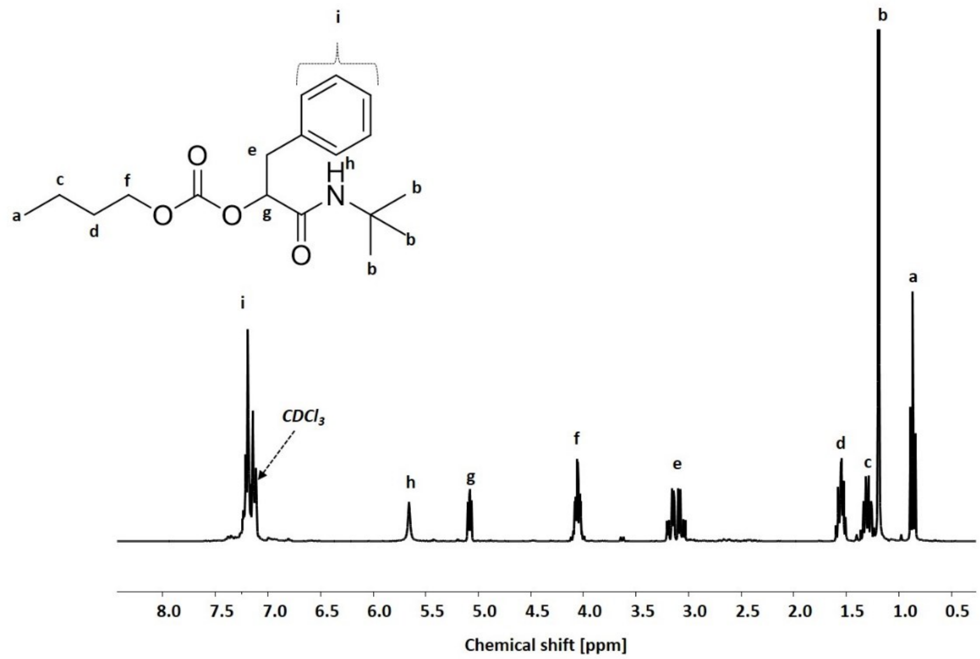

***Figure SI 10*a**: ^{1}H ($CDCl_3$) NMR of P-4CR **1b.**

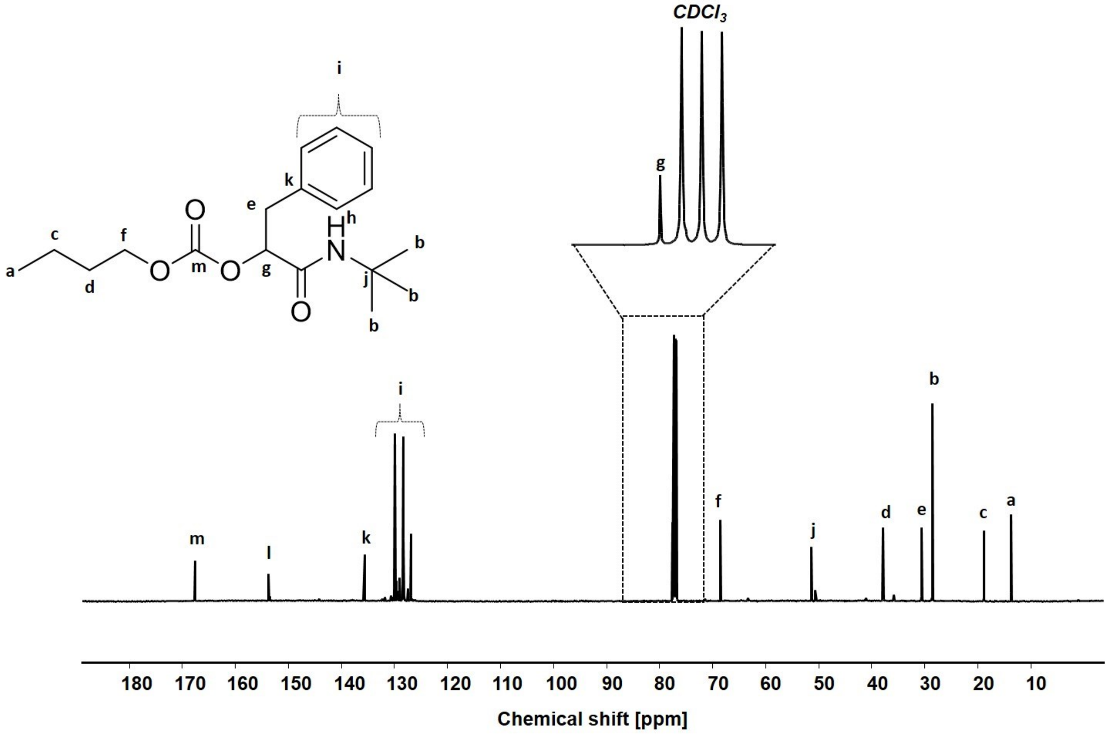

Figure SI 10b: ^{13}C ($CDCl_3$) NMR of P-4CR **1b.**

Molecule 1c. Butyl(1-(cyclohexylamino)-3-methyl-1-oxobutan-2-yl) carbonate

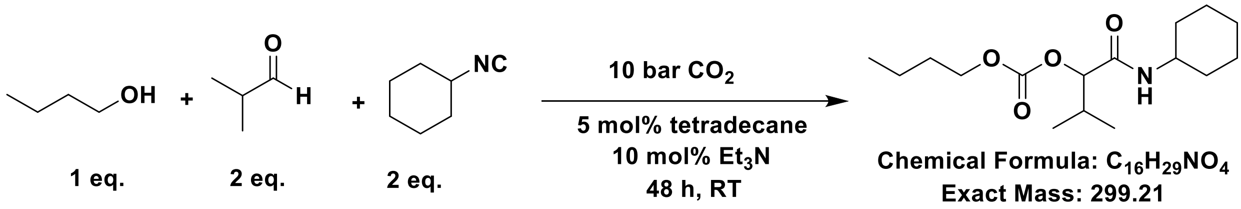

0.41 g of butanol (1 eq., 5.50 mmol) and 5 mol% tetradecane standard (70 µL) were stirred in 1.5 mL of dichloromethane (DCM) at room temperature for few minutes, after which a sample was collected for GC analysis (T_0). 10 mol% triethylamine catalyst (80 µL) was added and the mixture was saturated with CO_2 (5 bar) for 15 minutes. In a similar fashion, 0.79 g of isobutyraldehyde (2 eq., 11 mmol, 1.0 mL) was pre-saturated with CO_2 (5 bar) for 10 minutes after which both solutions were mixed and further saturated with 5 bar of CO_2 between 10-15 minutes. Furthermore, 1.20 g of cyclohexyl isocyanide (2 eq., 11 mmol, 1.38 mL) was added and the reaction was performed under 10 bar of CO_2 for 48 h at room temperature (22-24 °C). After 48 h, a sample was collected for GC analysis (T_f) in order to calculate the conversion and the relative ratio values between the observed products. The crude mixture was concentrated via rotary evaporation and purified via column chromatography (gradient CH:EE starting from 15:1 to 5:1). The product was obtained as white powder. Conversion: 56%, isolated yield: 33%. The hydrolysis product was isolated in a yield of 30%.

^{1}H NMR (500 MHz, $CDCl_3$) δ (ppm) = 5.95 (d, NH, 1H), 4.94 (d, CH, 1H), 4.18 (t, CH_2, 2H), 3.79 (m, CH, 1H), 2.31 (m, CH, 1H), 1.89 (m, CH_2, 2H), 1.72-1.27 (m, 5 CH_2, 10H), 1.12 (m, 2 CH_2, 4H), 0.95 (dd, 3CH_3, 9H).

^{13}C NMR (125 MHz, $CDCl_3$) δ (ppm) = 168.15 (j), 154.62 (i), 81.73 (g), 68.82 (f), 48.01 (e), 33.26, 33.02, 30.88, 30.69 (d + c), 25.57, 24.89 (b), 19.00, 18.68, 16.50, 13.76 (a).

ATR-IR: ν (cm^{-1}) = 3292.6, 3082.6, 2968.5, 2926.1, 2876.1, 2854.5, 1746.5, 1655.6, 1555.5, 1243.4, 1143.7, 784.5, 656.0.

Exact mass: $[C_{16}H_{29}NO_4Na]^+$ = 322.21 gmol^{-1}, obtained (ESI-MS) = 322.20 gmol^{-1}

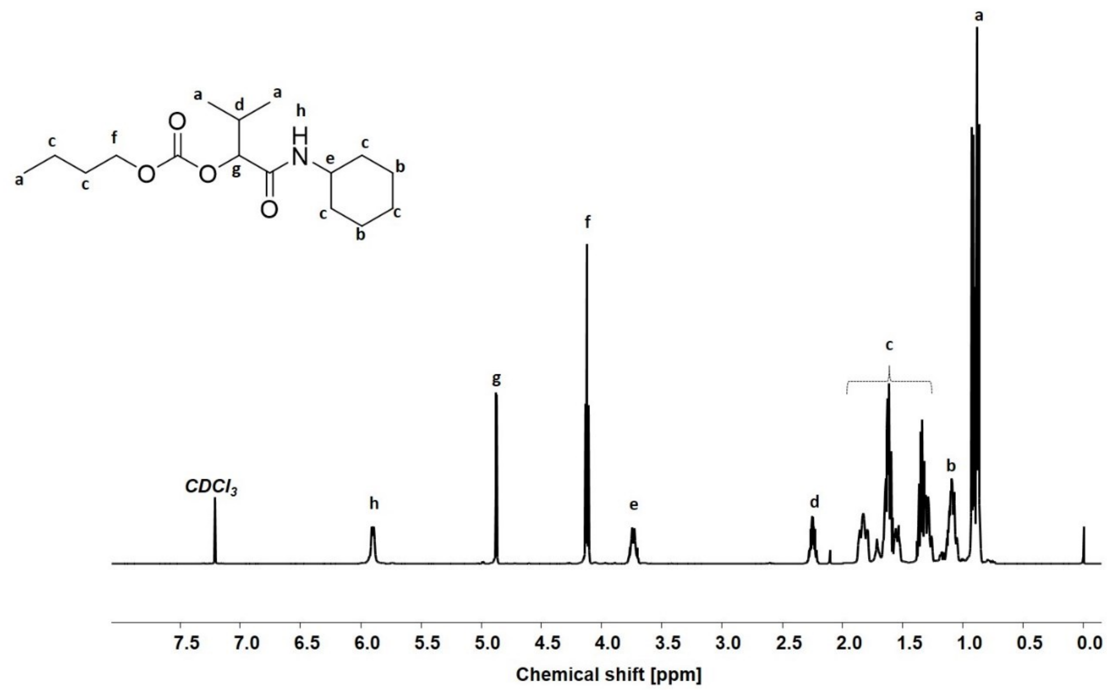

Figure SI 11**a**: ^{1}H ($CDCl_3$) NMR of P-4CR **1c.**

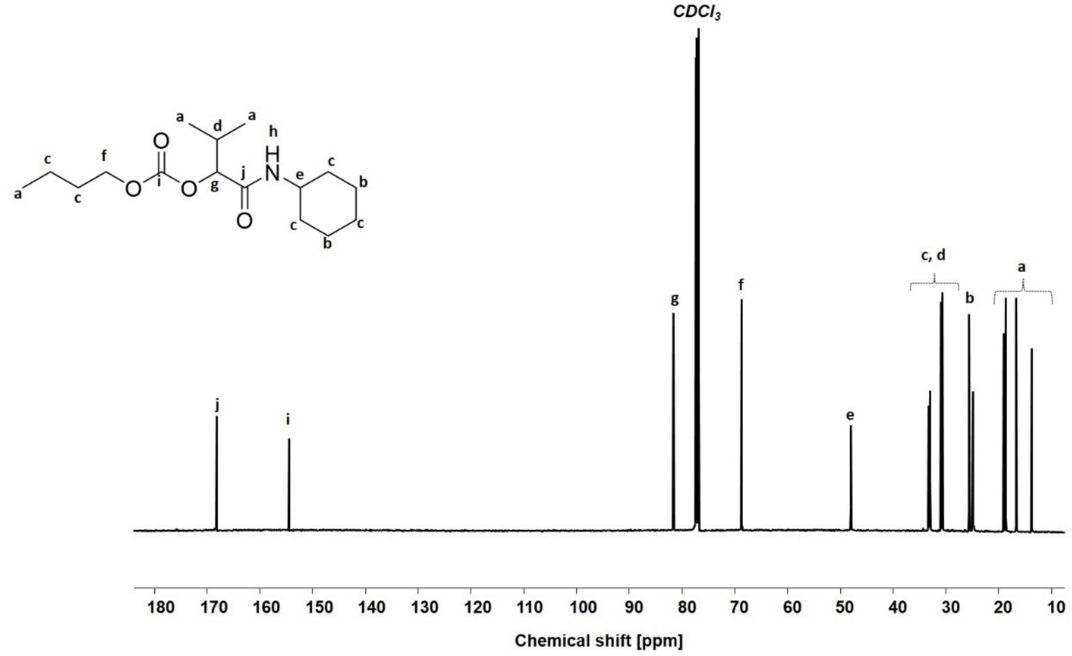

Figure SI 11**b**: ^{13}C ($CDCl_3$) NMR of P-4CR **1c.**

Molecule 2a. *sec*-Butyl (1-(tert-butylamino)-3-methyl-1-oxobutan-2-yl) carbonate

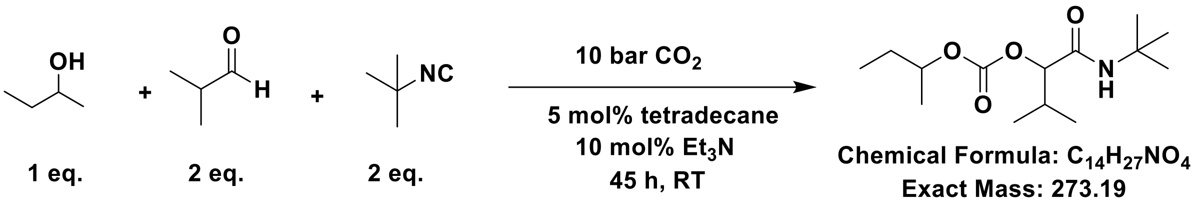

0.41 g of 2-butanol (1 eq., 5.50 mmol) and 5 mol% tetradecane standard (70 µL) were stirred in 1.5 mL of dichloromethane (DCM) at room temperature for 1 to 2 minutes, after which a sample was collected for GC analysis (T_0). 10 mol% triethylamine catalyst (80 µL) was added and the mixture was saturated with CO_2 (5 bar) for 15 minutes. In a similar fashion, 0.79 g of isobutyraldehyde (2 eq., 11 mmol, 1.0 mL) was pre-saturated with CO_2 (5 bar) for 10 minutes after which both solutions were mixed and further saturated with 5 bar of CO_2 between 10 to 15 minutes. Furthermore, 0.91 g of *tert*-butyl isocyanide (2 eq., 11 mmol, 1.24 mL) was added and the reaction was performed under 10 bar of CO_2 for 45 h at room temperature (22-24 °C). After 45 h, a sample was collected for GC analysis (T_f) in order to calculate the conversion and the relative ratio values between the observed products. The crude mixture was concentrated via rotary evaporation and purified via column chromatography (gradient CH:EE starting from 15:1 to 5:1). The product was obtained as white powder. Conversion: 54%, isolated yield: 24%.

^{1}H NMR (500 MHz, $CDCl_3$) δ (ppm) = 5.87 (s, NH, 1H), 4.82 (d, CH, 1H), 4.72 (m, CH, 1H), 2.28 (m, CH, 1H), 1.63 (m, CH_2, 2H), 1.34 (dt, 3 CH_3, 9H), 1.28 (t, CH_3, 3H), 0.94 (dt, 3 CH_3, 9H).

^{13}C NMR (125 MHz, $CDCl_3$) δ (ppm) = 168.46 (k), 154.31 (j), 81.60 (g), 77.53 (f), 51.40 (i), 30.75 (e), 29.02 (d + c), 19.54, 19.49, 18.70, 16.81 (b + a), 9.89 (a).

ATR-IR: ν (cm^{-1}) = 3307.2, 3092.8, 2966.6, 2933.1, 2875.2, 1741.6, 1663.4, 1560.9, 1360.2, 1247.0, 1108.4, 971.4, 888.3, 648.2, 402.2

Exact mass: $[C_{14}H_{27}NO_4H]^+$ = 274.19 $gmol^{-1}$, obtained (ESI-MS) = 274.20 $gmol^{-1}$

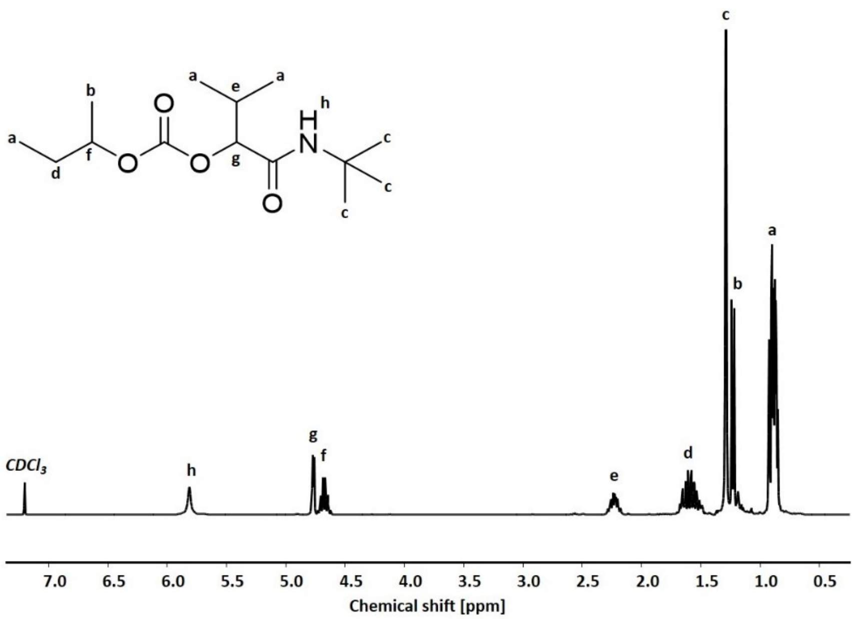

***Figure SI 12*a**: ^{1}H ($CDCl_3$) NMR of P-4CR **2a.**

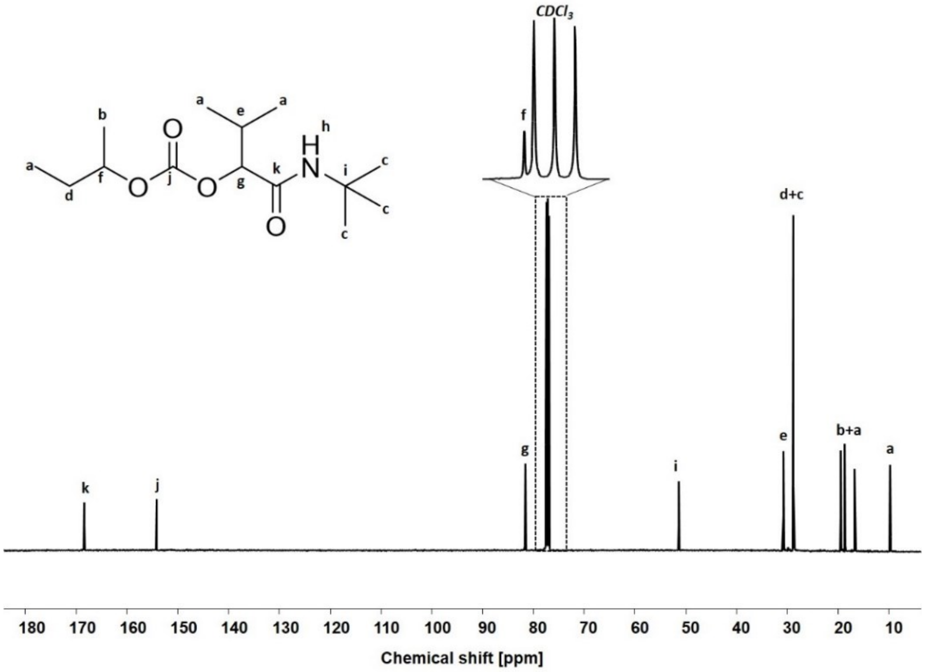

***Figure SI 12*b**: ^{13}C ($CDCl_3$) NMR of P-4CR **2a.**

Molecule 3a. Allyl (1-(tert-butylamino)-3-methyl-1-oxobutan-2-yl) carbonate

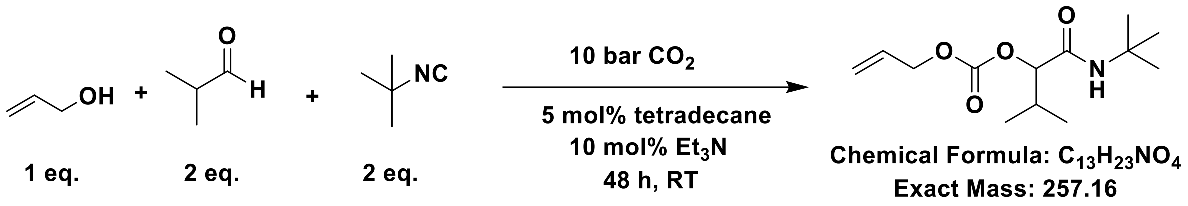

0.32 g of allyl-alcohol (1 eq., 5.50 mmol) and 5 mol% tetradecane standard (70 µL) were stirred in 1.5 mL of dichloromethane (DCM) at room temperature for 1 to 2 minutes, after which a sample was collected for GC analysis (T_0). 10 mol% triethylamine catalyst (80 µL) was added and the mixture was saturated with CO_2 (5 bar) for 15 minutes. In a similar fashion, 0.79 g of isobutyraldehyde (2 eq., 11 mmol, 1.0 mL) was pre-saturated with CO_2 (5 bar) for 10 minutes after which both solutions were mixed and further saturated with 5 bar of CO_2 between 10 to 15 minutes. Furthermore, 0.91 g of *tert*-butyl isocyanide (2 eq., 11 mmol, 1.24 mL) was added and the reaction was performed under 10 bar of CO_2 for 48 h at room temperature (22-24 °C). After 48 h, a sample was collected for GC analysis (T_f) in order to calculate the conversion and the relative ratio values between the observed products. The crude mixture was concentrated via rotary evaporation and purified via column chromatography (gradient CH:EE starting from 15:1 to 5:1). A second column (1:1, cyclohexane:diethyl ether) was necessary to separate the P-3CR side product from the expected P-4-CR product. The product was obtained as white powder. Conversion: 82%, isolated yield: 33%. The hydrolysis product was isolated with a yield of 23%.

^{1}H NMR (500 MHz, $CDCl_3$) δ (ppm) = 6.01-5.79 (m, NH + =CH, 2H), 5.36 (m, =CH_2, 2H), 4.85 (d, CH, 1H), 4.67 (dt, CH_2, 2H), 2.28 (m, CH, 1H), 1.35 (s, 3 CH_3, 9H), 0.92 (m, 2 CH_3, 6H).

^{13}C NMR (125 MHz, $CDCl_3$) δ (ppm) = 168.17 (k), 154.35 (j), 131.35 (g), 119.77 (f), 82.00 (e), 69.10 (d), 51.52 (i), 31.08 (c), 28.82 (b), 18.77, 16.94 (a).

ATR-IR: ν (cm^{-1}) = 3306.6, 3092.7, 2967.2, 2935.8, 2880.3, 1748.4, 1661.8, 1561.6, 1367.3, 1297.1, 1239.6, 979.4, 789.6, 657.1, 403.5.

Exact mass: $[C_{13}H_{23}NO_4Na]^+$ = 280.16 $gmol^{-1}$, obtained (ESI-MS) = 280.15 $gmol^{-1}$

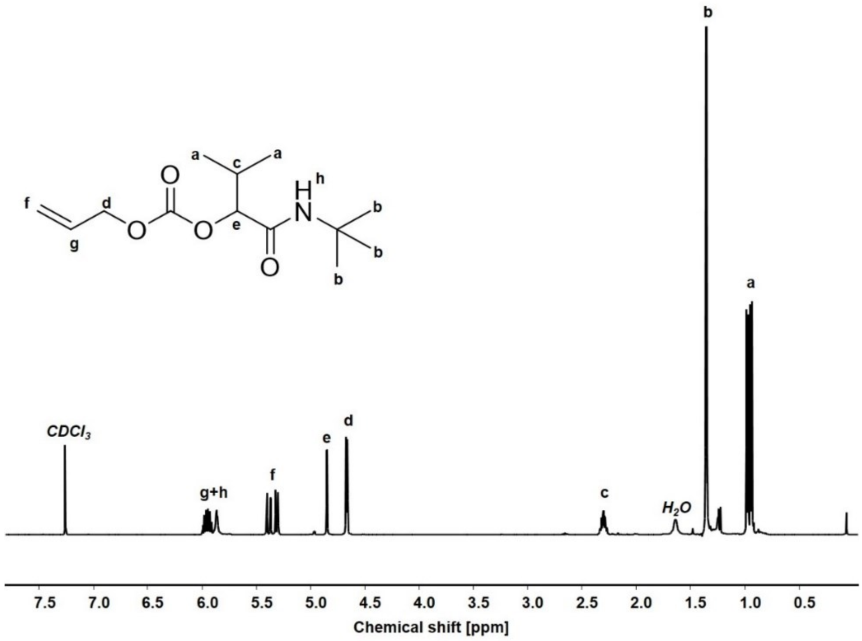

***Figure SI 13*a**: ^{1}H ($CDCl_3$) NMR of P-4CR **3a.**

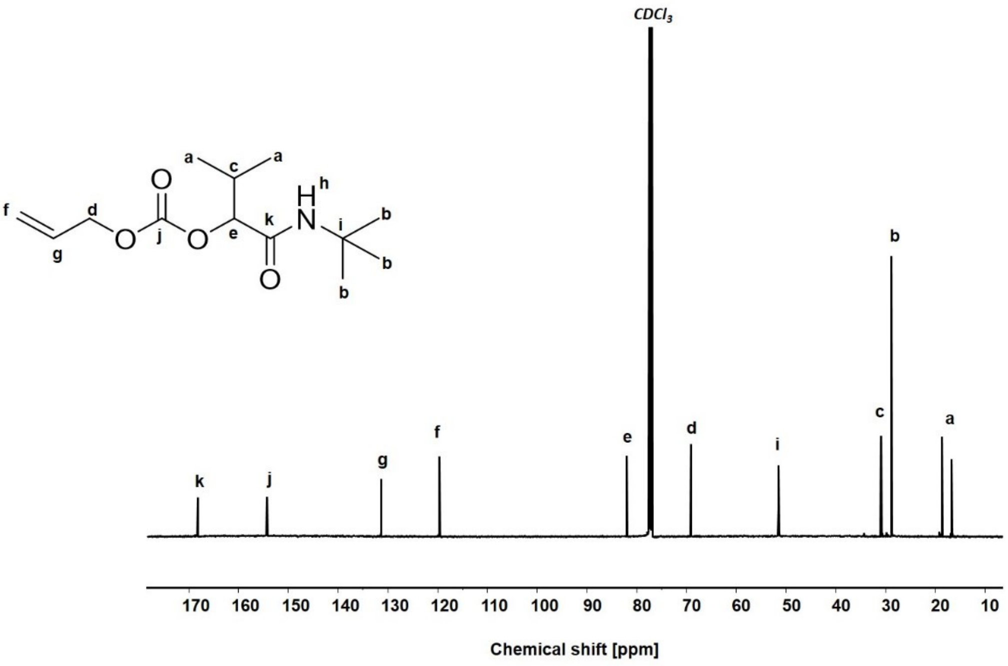

***Figure SI 13*b**: ^{13}C ($CDCl_3$) NMR of P-4CR **3a.**

Molecule 4a. Benzyl (1-(tert-butylamino)-3-methyl-1-oxobutan-2-yl) carbonate

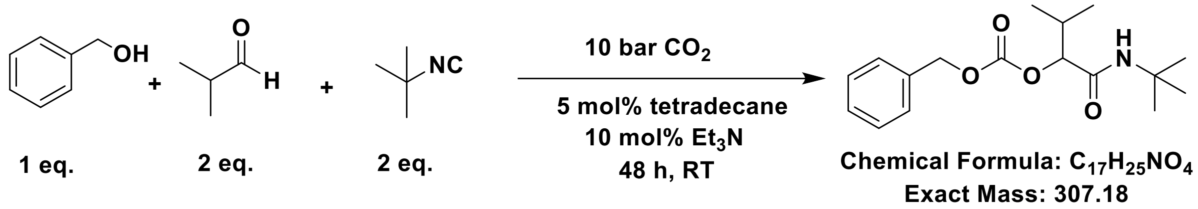

0.60 g of benzyl alcohol (1 eq., 5.50 mmol) and 5 mol% tetradecane standard (70 µL) were stirred in 1.5 mL of dichloromethane (DCM) at room temperature for 1 to 2 minutes, after which a sample was collected for GC analysis (T_0). 10 mol% triethylamine catalyst (80 µL) was added and the mixture was saturated with CO_2 (5 bar) for 15 minutes. In a similar fashion, 0.79 g of isobutyraldehyde (2 eq., 11 mmol, 1.0 mL) was pre-saturated with CO_2 (5 bar) for 10 minutes after which both solutions were mixed and further saturated with 5 bar of CO_2 between 10 to 15 minutes. Furthermore, 0.91 g of *tert*-butyl isocyanide (2 eq., 11 mmol, 1.24 mL) was added and reaction performed under 10 bar of CO_2 for 48 h at room temperature (22-24 °C). After 48 h, a sample was collected for GC analysis (T_f) in order to calculate the conversion and the relative ratio values between the observed products. The crude mixture was concentrated via rotary evaporation and purified via column chromatography (gradient CH:EE starting from 15:1 to 5:1). The product was obtained as white powder. Conversion: 33%, isolated yield: 27%.

^{1}H NMR (500 MHz, $CDCl_3$) δ (ppm) = 7.38 (m, Ar-H, 5H), 5.82 (s, NH, 1H), 5.20 (d, CH_2, 2H), 4.82 (d, CH, 1H), 2.28 (m, CH, 1H), 1.31 (s, 3 CH_3, 9H), 0.94 (dt, 2 CH_3, 6H).

^{13}C NMR (125 MHz, $CDCl_3$) δ (ppm) = 168.14 (k), 154.38 (j), 135.00 (i), 128.94, 128.85, 128.61 (g), 82.12 (d), 70.32 (e), 51.46 (h), 30.85 (c), 28.76 (b), 18.65, 16.72 (a).

ATR-IR: ν (cm^{-1}) = 3310.6, 3090.9, 3035.6, 2966.8, 2934.3, 2880.9, 1752.3, 1656.2, 1556.7, 1299.1, 1255.0, 1224.5, 986.3, 733.2, 654.0, 482.9.

Exact mass: $[C_{17}H_{25}NO_4Na]^+$ = 330.18 $gmol^{-1}$, obtained (ESI-MS) = 330.17 $gmol^{-1}$

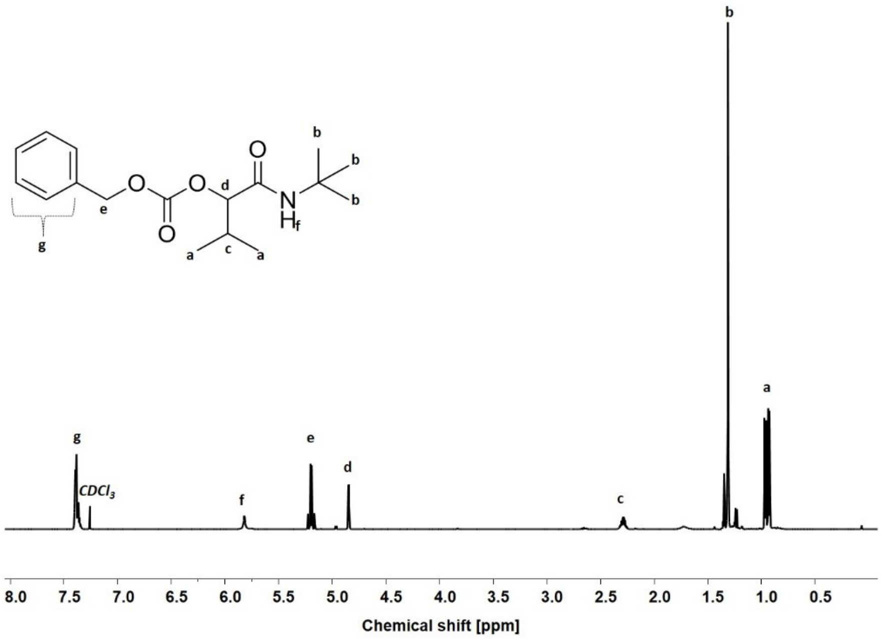

***Figure SI 14*a**: ^{1}H ($CDCl_3$) NMR of P-4CR **4a.**

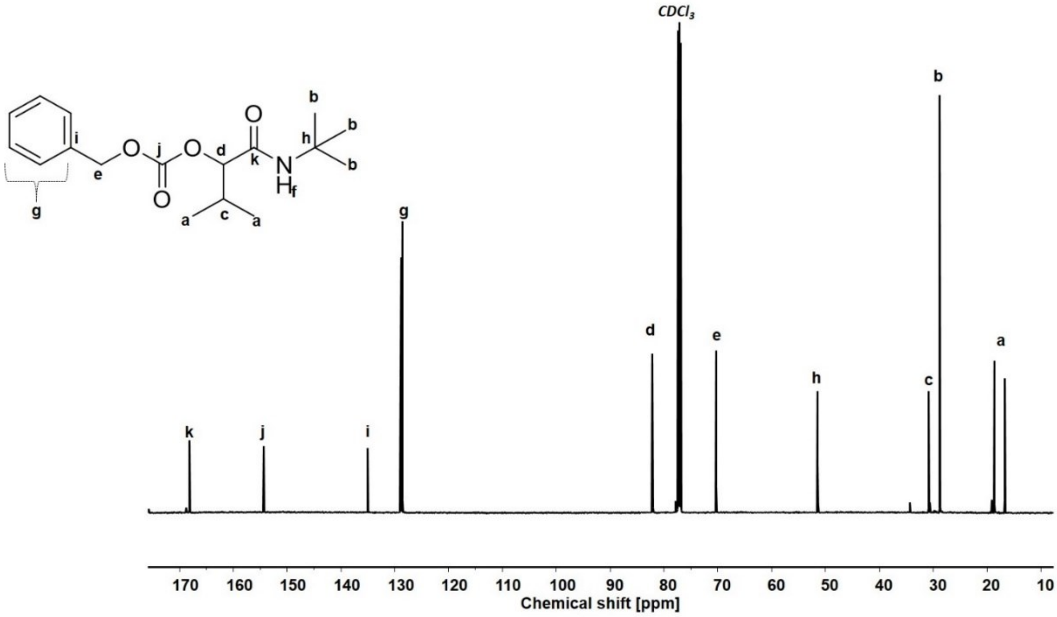

***Figure SI 14*b**: ^{13}C ($CDCl_3$) NMR of P-4CR **4a.**

Molecule 5a. Octyl (1-(tert-butylamino)-3-methyl-1-oxobutan-2-yl) carbonate

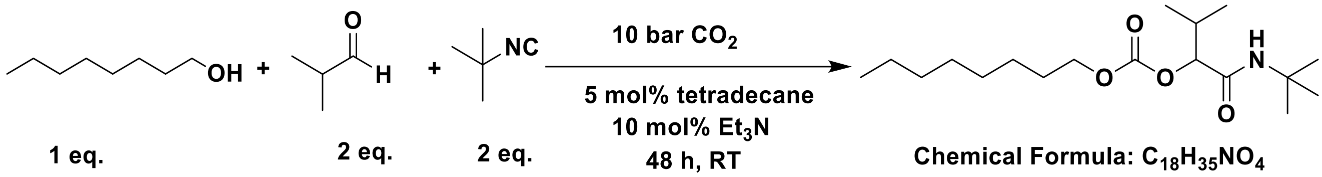

Chemical Formula: $C_{18}H_{35}NO_4$
Exact Mass: 329.26

0.72 g of 1-octanol (1 eq., 5.50 mmol) and 5 mol% tetradecane standard (70 µL) were stirred in 1.5 mL of dichloromethane (DCM) at room temperature for 1 to 2 minutes, after which a sample was collected for GC analysis (T_0). 10 mol% triethylamine catalyst (80 µL) was added and the mixture was saturated with CO_2 (5 bar) for 15 minutes. In a similar fashion, 0.79 g of isobutyraldehyde (2 eq., 11 mmol, 1.0 mL) was pre-saturated with CO_2 (5 bar) for 10 minutes after which both solutions were mixed and further saturated with 5 bar of CO_2 between 10-15 minutes. Furthermore, 0.91 g of *tert*-butyl isocyanide (2 eq., 11 mmol, 1.24 mL) was added and the reaction was performed under 10 bar of CO_2 for 48 h at room temperature (22-24 °C). After 48 h, a sample was collected for GC analysis (T_f) in order to calculate the conversion and the relative ratio values between the observed products. The crude mixture was concentrated via rotary evaporation and purified via column chromatography (gradient CH:EE starting from 15:1 to 5:1). The product was obtained as a viscous liquid. Conversion: 26%, isolated yield: 25%.

^{1}H NMR (500 MHz, $CDCl_3$) δ (ppm) = 5.87 (s, NH, 1H), 4.84 (d, CH, 1H), 4.17 (t, CH_2, 2H), 2.29 (m, CH, 1H), 1.71 (m, CH_2, 2H), 1.29 (m, 5 CH_2 + 3 CH_3 19H), 0.92 (m, 3 CH_3, 9H).

^{13}C NMR (125 MHz, $CDCl_3$) δ (ppm) = 168.34 (j), 154.54 (i), 81.76 (f), 68.95 (e), 51.47 (h), 31.87, 30.87, 29.28, 28.82, 25.76, 22.75, 18.68, 16.73, 14.21 (d + c + b + a).

ATR-IR: ν (cm^{-1}) =3329.5, 2962.5, 2926.5, 2873.4, 2855.7, 1749.3, 1665.9, 1521.8, 1453.5, 1247.2, 977.4, 787.0.

Exact mass: $[C_{18}H_{35}NO_4Na]^+$ = 352.26 $gmol^{-1}$, obtained (ESI-MS) = 352.25 $gmol^{-1}$

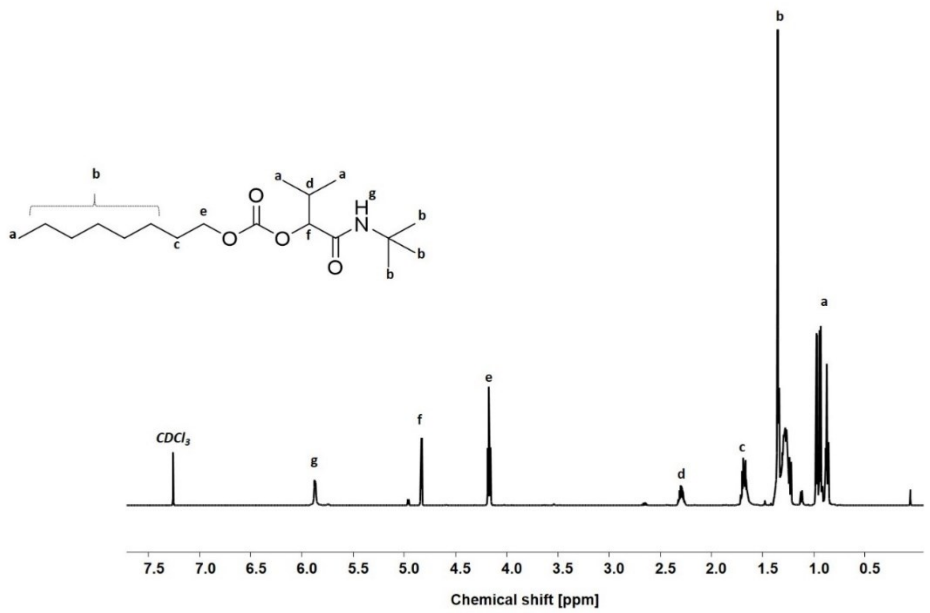

***Figure SI 15*a**: ^{1}H ($CDCl_3$) NMR of P-4CR **5a.**

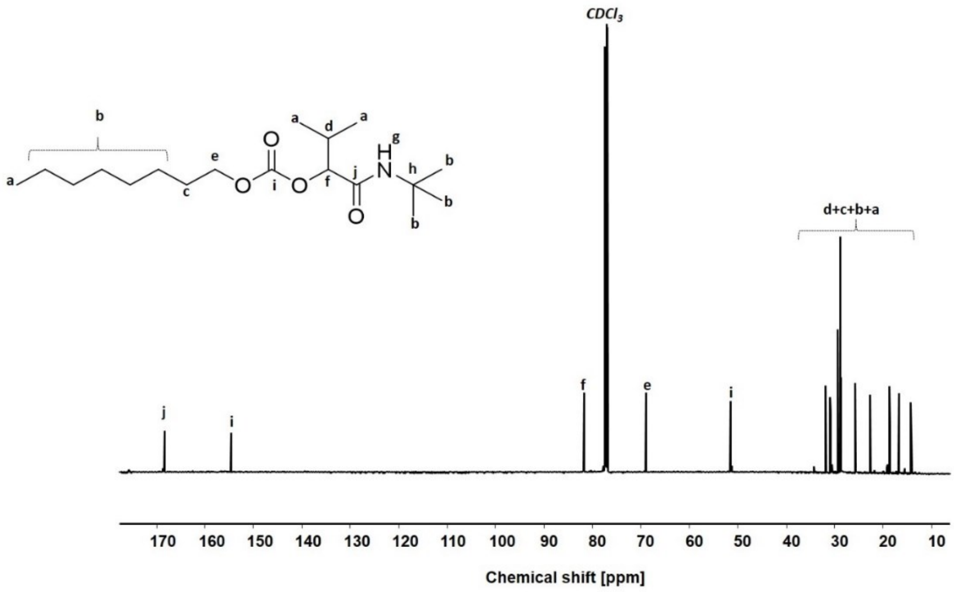

***Figure SI 15*b**: ^{13}C ($CDCl_3$) NMR of P-4CR **5a.**

Molecule 6a. Butyl (1-(cyclohexylamino)-1-oxododec-11-en-2-yl) carbonate

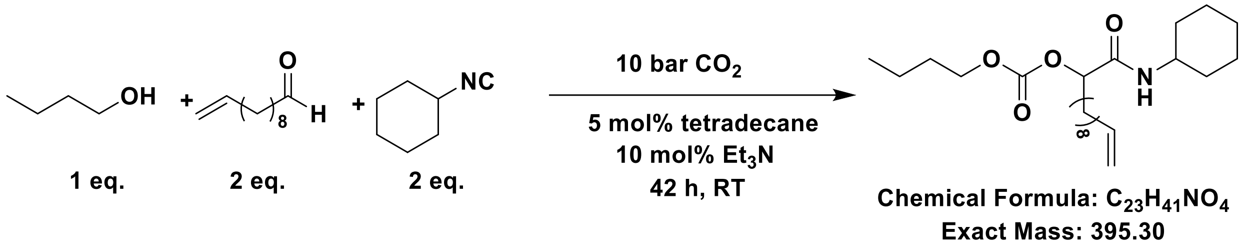

0.41 g of butanol (1 eq., 5.50 mmol) and 5 mol% tetradecane standard (70 µL) were stirred in 1.5 mL of dichloromethane (DCM) at room temperature for 1 to 2 minutes, after which a sample was collected for GC analysis (t_0). 10 mol% Triethylamine catalyst (80 µL) was added and the mixture was saturated with CO_2 (5 bar) for 15 minutes. Next, 1.86 g of undecylenic aldehyde (2 eq., 11 mmol, 2.28 mL) was added, after which the solutions were mixed and further saturated with 5 bar of CO_2 for ~15 minutes. Furthermore, 1.21 g of cyclohexyl isocyanide (2 eq., 11 mmol, 1.38 mL) was added and the reaction performed under 10 bar of CO_2 for 42 h at room temperature (22-24 °C). After 42 h, a sample was collected for GC analysis (t_f) in order to calculate the conversion. The crude mixture was concentrated via rotary evaporation and purified via column chromatography (gradient CH:EE starting from 15:1 to 5:1). The product was obtained as white powder. Conversion: 30%, isolated yield: 43%.

^{1}H NMR (500 MHz, $CDCl_3$) δ (ppm) = 6.00 (d, NH, 1H), 5.80 (m, CH, 1H), 5.01-4.93 (m, HC=CH_2 ,3H), 4.18 (t, CH_2, 2H), 3.77 (m, CH, 1H), 2.02 (q, CH_2, 2H), 1.87-1.14 (m, 14 CH_2, 28H), 0.94 (t, 3 CH_3, 9H).

^{13}C NMR (125 MHz, $CDCl_3$) δ (ppm) = 168.62 (j), 154.12 (i), 139.29 (f2), 114.19 (f1), 77.60 (g), 68.60 (e), 48.00 (d), 33.91, 33.01, 32.10, 30.65, 29.38, 29.26, 29.11, 28.95, 25.51, 24.85, 24.49, 18.96 (c + d), 13.71 (a).

ATR-IR: ν (cm^{-1}) = 3287.6, 3077.5, 2915.8, 2848.0, 1745.7, 1654.8, 1556.9, 1246.1, 910.4, 696.3.

Exact mass: $[C_{23}H_{41}NO_4Na]^+$ = 418.30 $gmol^{-1}$, obtained (ESI-MS) = 418.29 $gmol^{-1}$

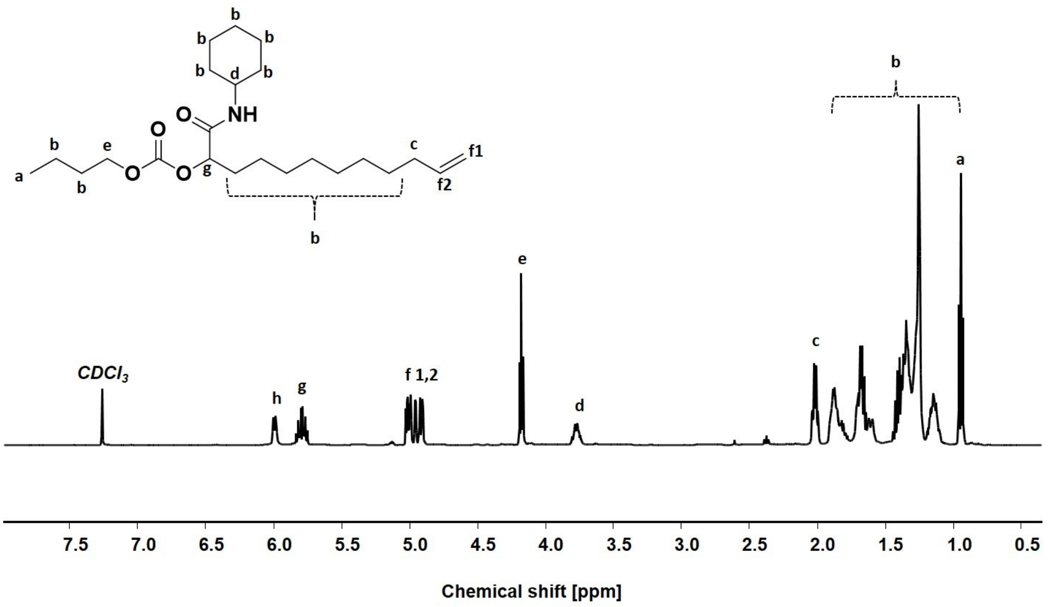

***Figure SI 16*a**: ^{1}H ($CDCl_3$) NMR of P-4CR **6a.**

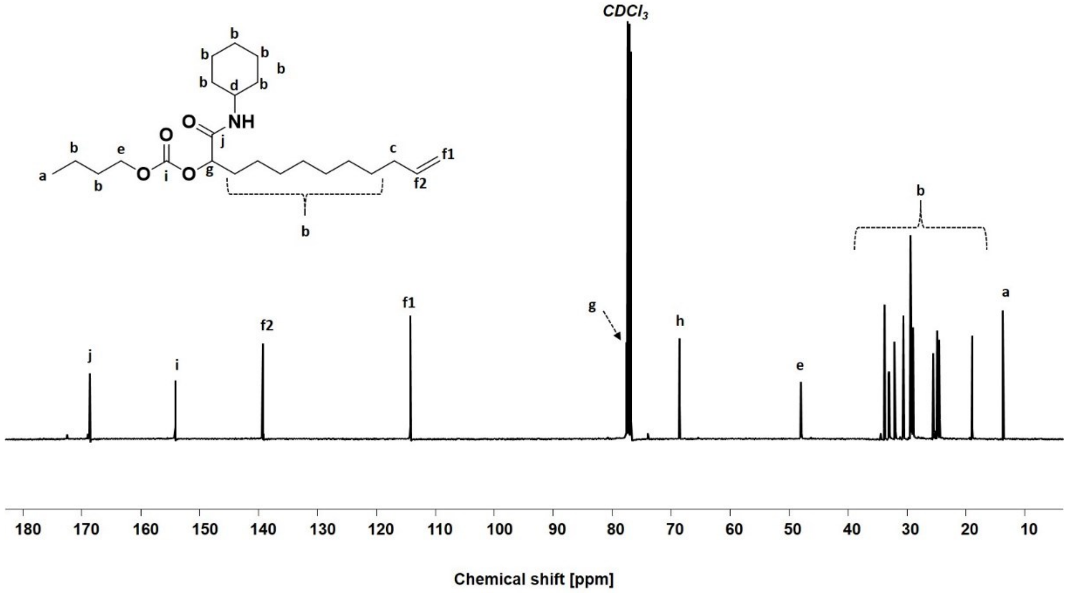

***Figure SI 16*b**: ^{13}C ($CDCl_3$) NMR of P-4CR **6a.**

Molecule 7a. Butyl (3,3-dimethyl-1-((2-morpholinoethyl)amino)-1-oxobutan-2-yl) carbonate

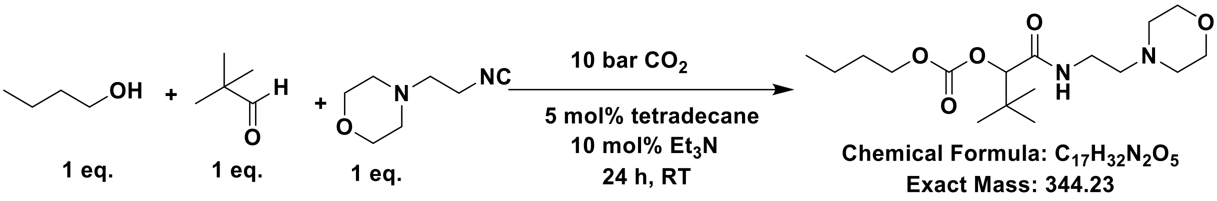

0.41 g of butanol (1 eq., 5.50 mmol) and 5 mol% tetradecane standard (70 µL) were stirred in 1.5 mL of dichloromethane (DCM) at room temperature for 1 to 2 minutes, after which a sample was collected for GC analysis (t_0). 10 mol% triethylamine catalyst (80 µL) was added and followed by applying of CO_2 (5 bar) for 15 minutes. Next, 0.48 g of trimethyl acetaldehyde (1 eq., 5.50 mmol, 0.61 mL) was pre-saturated with CO_2 (5 bar) for 15 minutes, mixed with the CO_2-saturated butanol solution and further stirred under 5 bar of CO_2 for 15 minutes. Finally, 0.78 g of 2-morpholinoethyl isocyanide (1 eq., 5.50 mmol, 0.76 mL) was added and the reaction performed under 10 bar of CO_2 for 24 h at room temperature (22-24 °C). After 24 h, a sample was collected for GC analysis (T_f) in order to calculate the conversion. The crude mixture was concentrated via rotary evaporation and purified via column chromatography (gradient CH:EE starting from 15:1 to 5:1). The product was obtained as a viscous liquid. Conversion: 19%, isolated yield: 24%.

^{1}H NMR (500 MHz, $CDCl_3$) δ (ppm) = 6.53 (br, NH, 1H), 4.70 (s, CH, 1H), 4.16 (t, CH_2, 2H), 3.43 (m, CH_2OCH_2, , 4H), 3.32 (m, CH_2NCO, 2H), 2.50 (m, 3 CH_2N, 6H), 1.67 (m, CH_2, 2H), 1.40 (m, CH_2, 2H), 1.04 (s, 3 CH_3, 9H), 0.94 (t, 3 CH_3, 9H).

^{13}C NMR (125 MHz, $CDCl_3$) δ (ppm) = 168.41 (m), 154.56 (l), 84.50 (i), 68.56 (h), 67.00 (g), 56.89, 53.32 (e), 35.31 (f), 34.38 (k), 30.69 (d), 26.17 (b), 18.97 (c), 13.73(a).

ATR-IR: ν (cm^{-1}) = 3328.3, 2960.3, 2860.6, 2805.4, 1746.1, 1666.9, 1525.3, 1454.0, 1393.1, 1366.9, 1247.0, 1117.2, 959.4, 794.1.

Exact mass: $[C_{17}H3_2N_2O_5H]^+$ = 345.23 $gmol^{-1}$, obtained (ESI-MS) = 345.24 $gmol^{-1}$

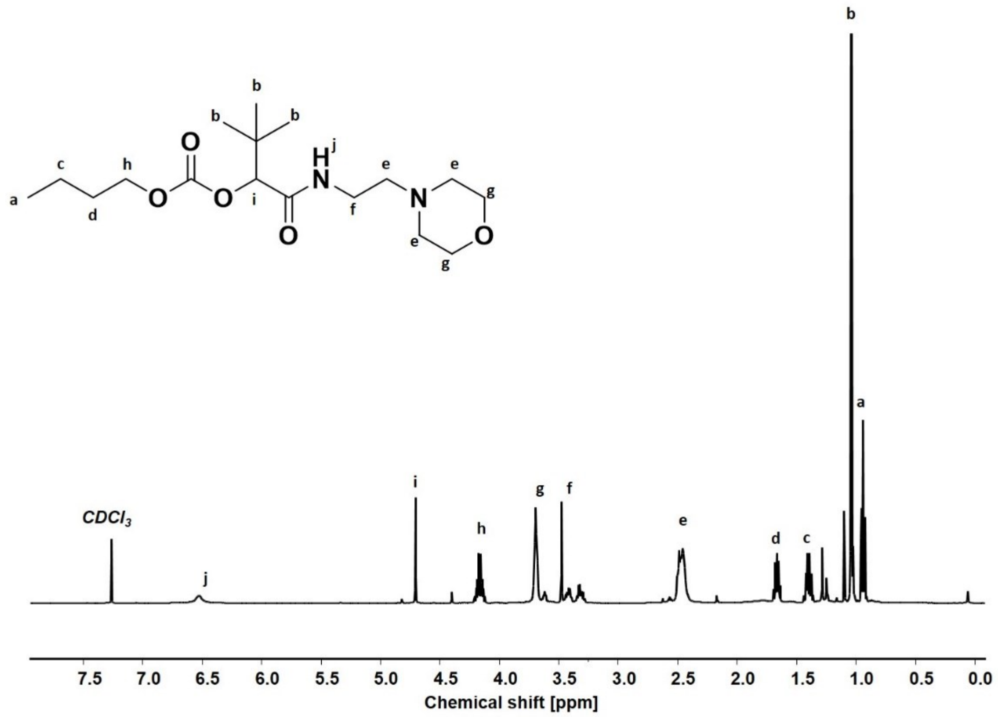

*Figure SI 17*a: ^{1}H ($CDCl_3$) NMR of P-4CR **7a.**

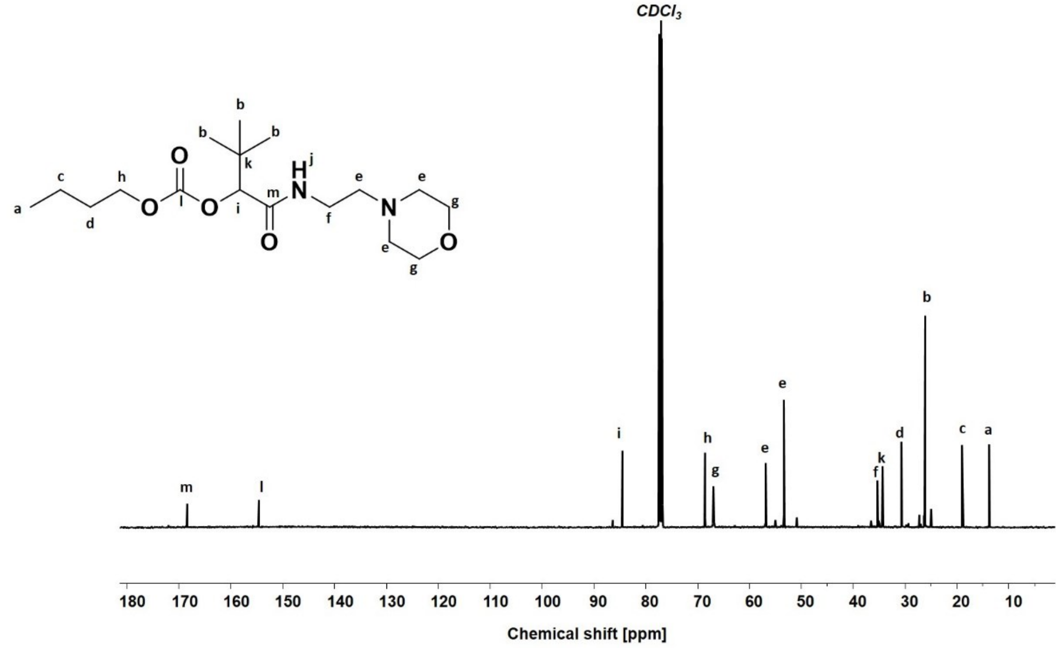

*Figure SI 17*b: ^{13}C ($CDCl_3$) NMR of P-4CR **7a.**

Molecule 8a. 1-(adamantan-1-ylamino)-1-oxododec-11-en-2-yl butyl carbonate

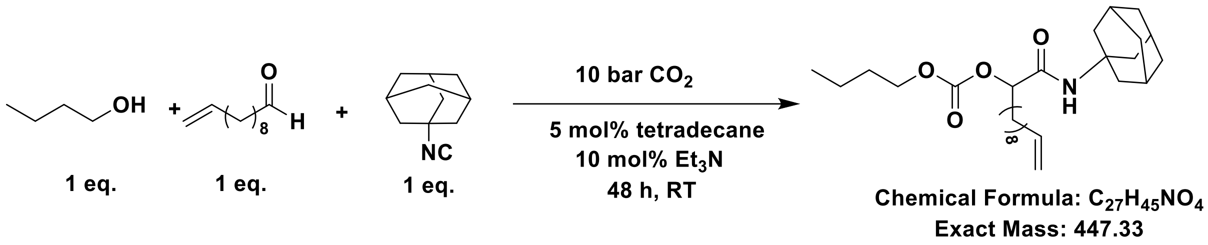

0.41 g of butanol (1 eq., 5.50 mmol) and 5 mol% tetradecane standard (70 µL) were stirred in 1.5 mL of dichloromethane (DCM) at room temperature for 1 to 2 minutes, after which a sample was collected for GC analysis (T_0). 10 mol% triethylamine catalyst (80 µL) was added and the mixture was saturated with CO_2 (5 bar) for 15 minutes. Next, 0.93 g of undecylenic aldehyde (1 eq., 5.50 mmol, 1.14 mL) was added, after which the solutions were mixed and further saturated with 5 bar of CO_2 for 10-15 minutes. Furthermore, 0.89 g of 1-adamantyl isocyanide (1 eq., 5.5 mmol) was added and the reaction performed under 10 bar of CO_2 for 48 h at room temperature (22-24 °C). After 48 h, a sample was collected for GC analysis (T_f) in order to calculate the conversion. The crude mixture was concentrated via rotary evaporation and purified via column chromatography (gradient CH:EE starting from 15:1 to 5:1). The product was obtained as white powder. Conversion: 16%, isolated yield: 24%.

^{1}H NMR (500 MHz, $CDCl_3$) δ (ppm) = 5.84-5.78 (m, NH + CH, 2H), 4.97-4.92 (e1 + e2, $CH=CH_2$, 3H), 4.18 (t, CH_2, 2H), 2.08-1.67 (m, 8 CH_2 + 3 CH, 19H), 1.36-1.26 (m, 8 CH_2, 16H), 0.95 (t, 3 CH_3, 9H).

^{13}C NMR (125 MHz, $CDCl_3$) δ (ppm) = 168.51 (j), 154.35 (i), 139.43 (e2), 114.34 (e1), 77.74 (f), 68.54 (d), 52.03 (h), 41.56, 36.35, 33.87, 32.04, 30.68, 29.47, 29.40, 29.30, 28.98, 24.48, 18.99 (c + b), 13.75 (a).

ATR-IR: ν (cm^{-1}) = 3309.9, 3081.3, 2907.0, 2845.1, 1747.7, 1657.4, 1552.0, 1228.9, 898.6, 630.4.

Exact mass: $[C_{27}H_{45}NO_4Na]^+$ = 470.33 $gmol^{-1}$, obtained (ESI-MS) = 470.32 $gmol^{-1}$

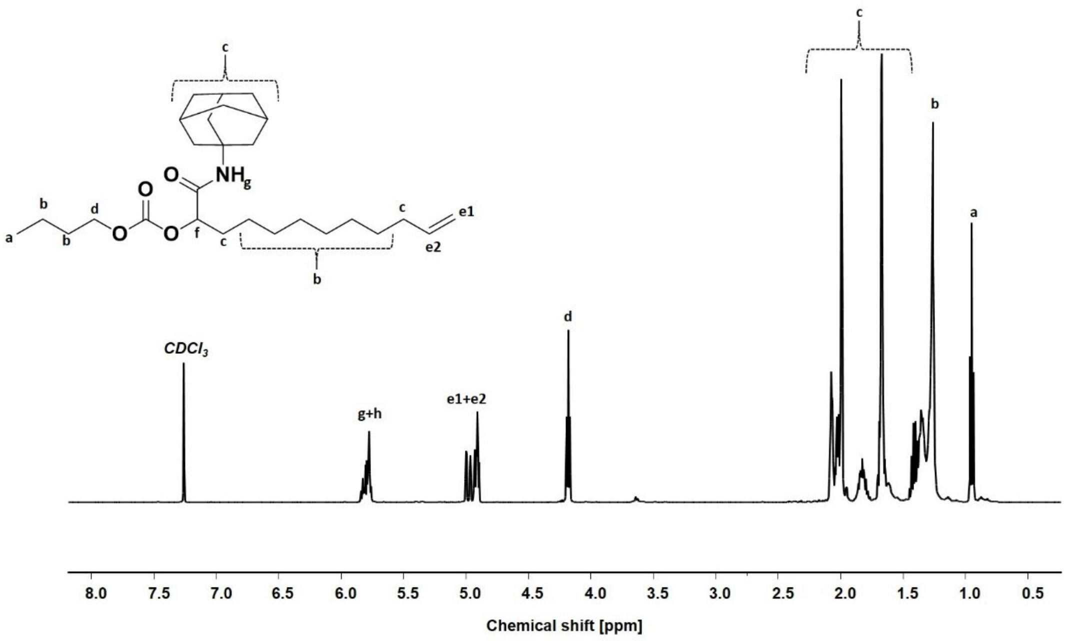

*Figure SI 18*a: ^{1}H ($CDCl_3$) NMR of P-4CR **8a.**

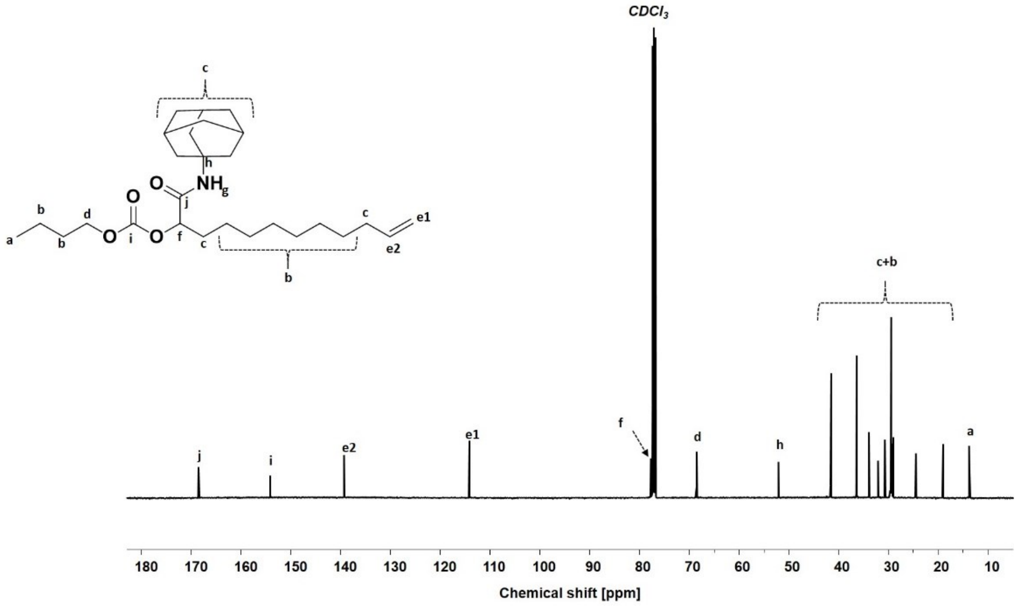

*Figure SI 18*b: ^{13}C ($CDCl_3$) NMR of P-4CR **8a.**

Molecule 9a. Butyl (2-(tert-butylamino)-1-(2,4-dinitrophenyl)-2-oxoethyl) carbonate

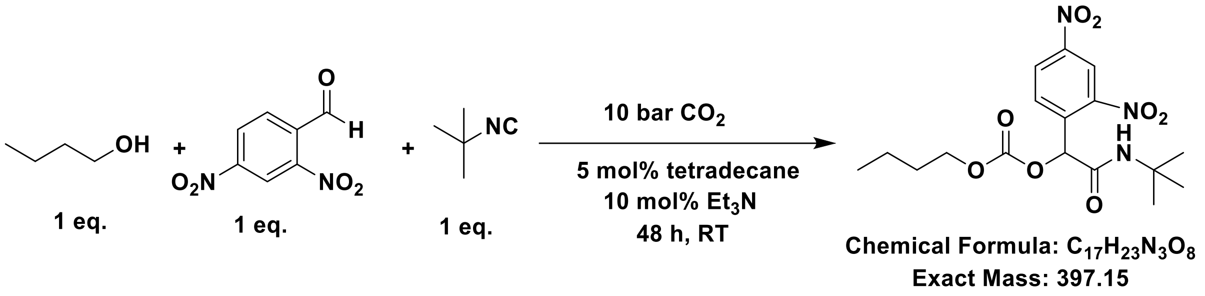

0.41 g of butanol (1 eq., 5.50 mmol) and 5 mol% tetradecane standard (70 µL) were stirred in 1.5 mL of dichloromethane (DCM) at room temperature for 1 to 2 minutes, after which a sample was collected for GC analysis (T_0). 10 mol% triethylamine catalyst (80 µL) was added and the mixture was saturated with CO_2 (5 bar) for 15 minutes. Next, 1.08 g of 2,4-dinitrobenzaldehyde (1 eq., 5.50 mmol) was added, allowed to solubilize in the butanol-DCM-CO_2 mixture and further saturated with 5 bar of CO_2 for ~15 minutes. Furthermore, 0.46 g of *tert* butyl isocyanide (1 eq., 5.50 mmol, 0.63 mL) was added and the reaction performed under 10 bar of CO_2 for 48 h at room temperature (22-24 °C). After 48 h, a sample was collected for GC analysis (T_f) in order to calculate the conversion and relative ratio values between the P-4CR and its hydrolysis product. The crude mixture was concentrated via rotary evaporation and purified via column chromatography (gradient CH:EE starting from 15:1 to 2:1). The product was obtained as white powder. Conversion: 40%, isolated yield: 22%.

^{1}H NMR (500 MHz, $CDCl_3$) δ (ppm) = 8.86 (d, Ar-CH, 1H), 8,47 (dd, Ar-CH, 1H), 7.93 (d, Ar-CH, 1H), 6.63 (s, CH, 1H), 6.29 (br, NH, 1H), 4.21 (m, CH_2, 2H), 1.66 (m, CH_2, 2H), 1.42-1.36 (m, 3 CH_3, + CH_2, 11H), 0.94 (t, 3 CH_3, 9H).

^{13}C NMR (125 MHz, $CDCl_3$) δ (ppm) = 164.18 (o), 153.75 (n), 148.38 (m), 147.85 (l), 137.32 (k), 130.73 (g), 127.54 (h), 120.47 (i), 73.92 (f), 69.60 (d), 52.35 (j), 30.52 (c), 28.61, 18.88 (b), 13.67 (a).

ATR-IR: ν (cm^{-1}) = 3415.4, 3113.2, 2969.0, 2932.2, 2862.5, 1755.1, 1668.2, 1532.3, 1345.6, 1248.8, 912.3, 735.5.

Exact mass: $[C_{17}H_{23}N_3O_8Na]^+$ = 420.15 $gmol^{-1}$, obtained (ESI-MS) = 420.14 $gmol^{-1}$

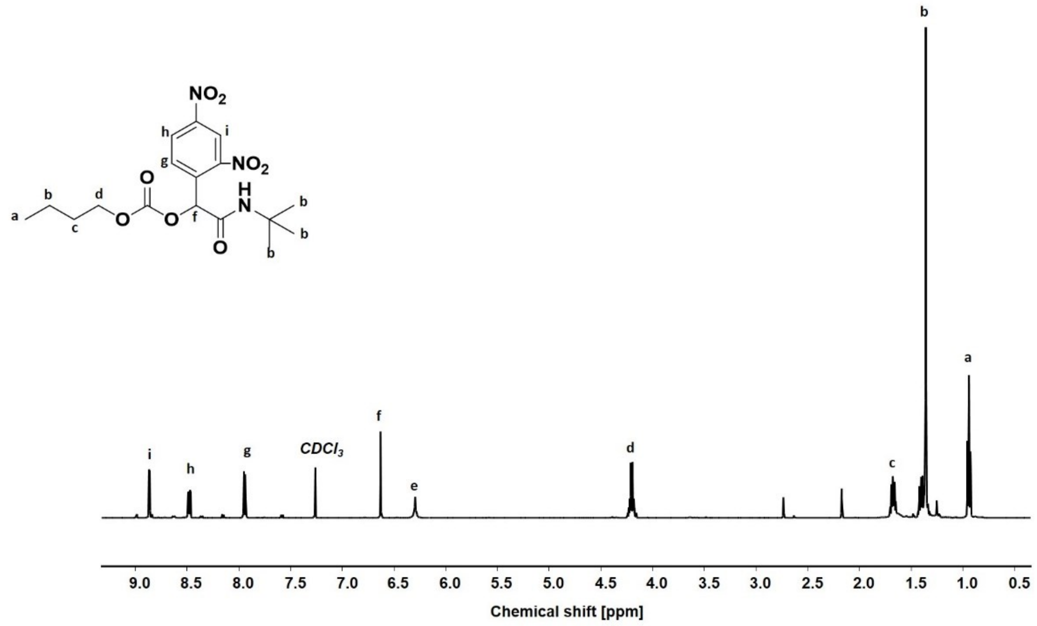

***Figure SI 19*a**: ^{1}H ($CDCl_3$) NMR of P-4CR **9a.**

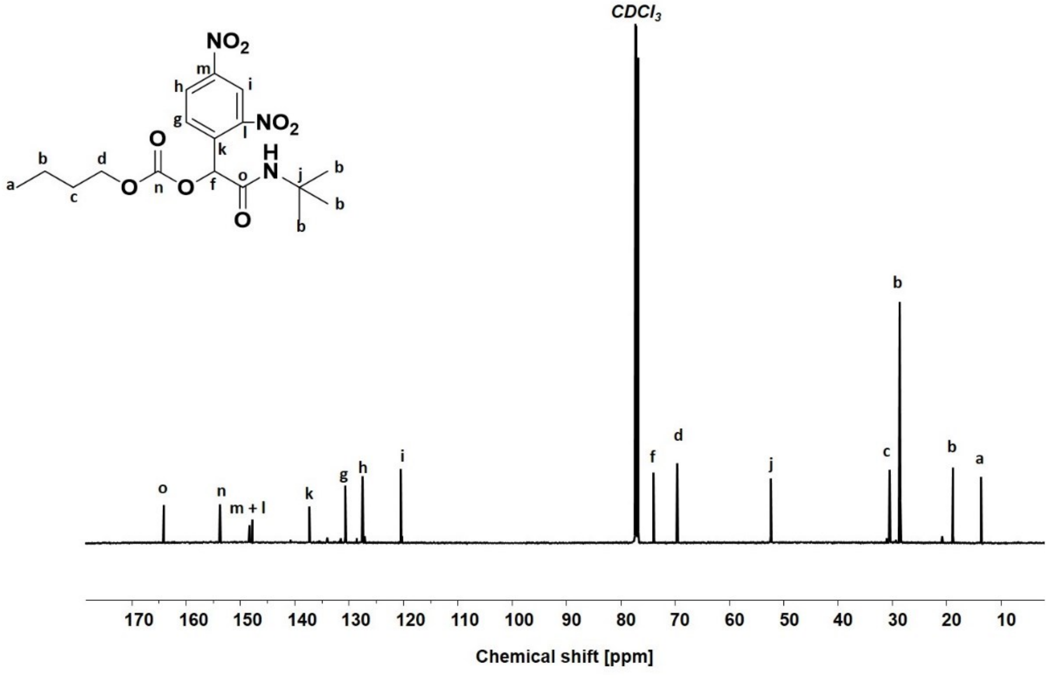

***Figure SI 19*b**: ^{13}C ($CDCl_3$) NMR of P-4CR **9a.**

Molecule 10a. 1-(tert-butylamino)-1-oxododec-11-en-2-yl cyclohexyl carbonate

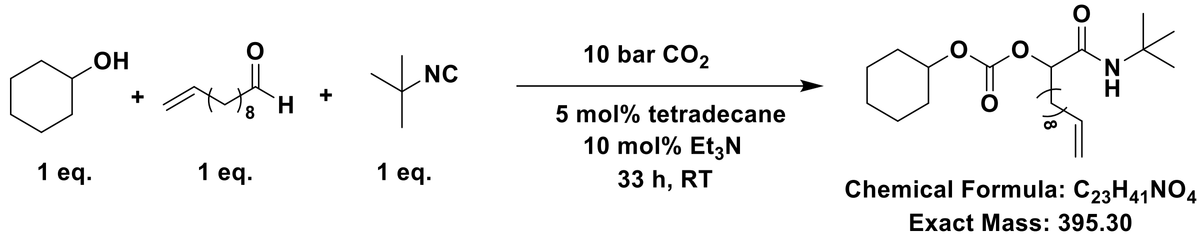

0.55 g of cyclohexanol (1 eq., 5.50 mmol) and 5 mol% tetradecane standard (70 µL) were stirred in 1.5 mL of dichloromethane (DCM) at room temperature for 1 to 2 minutes, after which a sample was collected for GC analysis (T_0). 10 mol% triethylamine catalyst (80 µL) was added and the mixture was saturated with CO_2 (5 bar) for 15 minutes. Next, 0.93 g of undecylenic aldehyde (1 eq., 5.50 mmol, 1.14 mL) was added, after which the solutions were mixed and further saturated with 5 bar of CO_2 between 10-15 minutes. Furthermore, 0.46 g of *tert* butyl isocyanide (1 eq., 5.50 mmol, 0.63 mL) was added and the reaction performed under 10 bar of CO_2 for 33 h at room temperature (22-24 °C). After 33 h, a sample was collected for GC analysis (T_f) in order to calculate the conversion and relative ratio values between observed products. The crude mixture was concentrated via rotary evaporation and purified via column chromatography (gradient CH:EE starting from 15:1 to 5:1). The product was obtained as sticky white powder. Conversion: 35%, isolated yield: 18%.

^{1}H NMR (500 MHz, $CDCl_3$) δ (ppm) = 5.92 (s, NH, 1H), 5.79 (m, CH, 1H), 4.98-4.88 (m, CH=CH_2, 3H), 4.61 (m, CH, 1H), 2.00-1.25 (m, 3 CH_3 + 13 CH_2, 35H).

^{13}C NMR (125 MHz, $CDCl_3$) δ (ppm) = 168.92 (h), 154.47 (g), 139.22 (c2), 114.16 (c1), 77.61 (b + d), 51.29 (f), 33.83, 31.89, 31.54, 29.35, 29.23, 29.09, 28.73, 26.94, 25.19, 24.52, 23.11 (a).

ATR-IR: ν (cm^{-1}) = 3315.68, 3074.56, 2923.50, 2860.56, 1752.74, 1663.65, 1520.34, 1454.49, 1245.32, 1015.82, 900.58, 794.06.

Exact mass: $[C_{23}H_{41}NO_4Na]^+$ = 418.30 $gmol^{-1}$, obtained (ESI-MS) = 418.29 $gmol^{-1}$

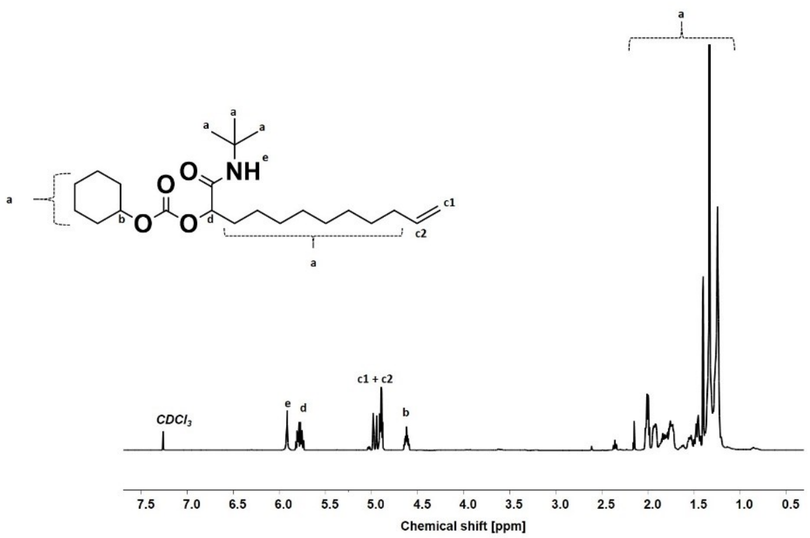

***Figure SI 20*a**: ^1H ($CDCl_3$) NMR of P-4CR **10a.**

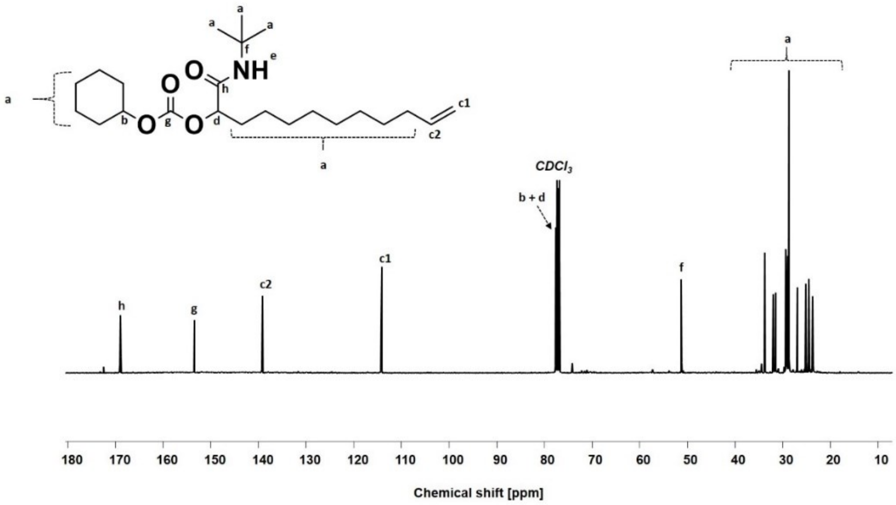

***Figure SI 20*b**: ^{13}C ($CDCl_3$) NMR of P-4CR **10b**

V. CHARACTERIZATION OF HYDROLYSIS PRODUCTS OF P-4CR

The obtained hydrolysis product of P-4CR products **1c** and **3c** were isolated and characterized. From the ^1H NMR spectra of the hydrolysis products of **1c** and **3a**, the amide proton chemical shifts were visible at 6.19 and 6.31 ppm, respectively. Also noticeable is the presence of the alcoholic proton chemical shift at 2.45 and 3.05 ppm, respectively. The presence of the amide bond is further confirmed from their ^{13}C NMR results. The carbonyl carbon of the amide gives a signal around 172.0 ppm for both compounds. In addition, from FT-IR of both compounds, we could attribute the sharp absorbance band around 3330 cm^{-1} to the N-H stretching band of the amide. This sharp peak overlaps with a broad signal at 3219 and 3258 cm^{-1} for the hydrolysis products of **1c** and **3a** respectively and attributed to the –OH absorbance band. Furthermore, the same amide functional group shows a C=O stretching absorbance band

around 1640 cm^{-1} in addition to the N-H bending absorbance band around 1530 cm^{-1} for both compounds. The C-O of their hydroxyl groups gives a stretching absorbance band around 1025 cm^{-1}. The masses of both compounds were equally confirmed from ESI-MS.

Hydrolysis product molecule 1c*:* *N*-(tert-butyl)-2-hydroxy-3-methylbutanamide

Isolated yield: 30%

^{1}H NMR (500 MHz, $CDCl_3$) δ (ppm) = 6.19 (s, NH, 1H), 3.94 (t, CH, 1H), 3.81 (m, CH, 1H), 2.45 (d, OH, 1H), 2.13 (m, CH, 1H), 1.91, 1.71, 1.62 (m, 3 CH_2, 6H), 1.37, 1.18 (m, 2 CH_2, 4H), 1.02, 0.85 (d, 2 CH_3, 6H).

^{13}C NMR (125 MHz, $CDCl_3$) δ (ppm) = 172.04 (i), 76.28 (g), 48.02 (f), 33.38, 33.24, 32.19 (d + c), 25.64, 24.94 (b), 19.26, 15.46 (a).

ATR-IR: ν (cm^{-1}) = 3326.3, 3219.0, 2927.6, 2852.3, 1631.9, 1533.3, 1451.0, 1023.7, 750.3.

Exact mass: $[C_{11}H_{21}NO_2Na]^+$ = 222.16 gmol^{-1}, obtained (ESI-MS) = 222.15 gmol^{-1}

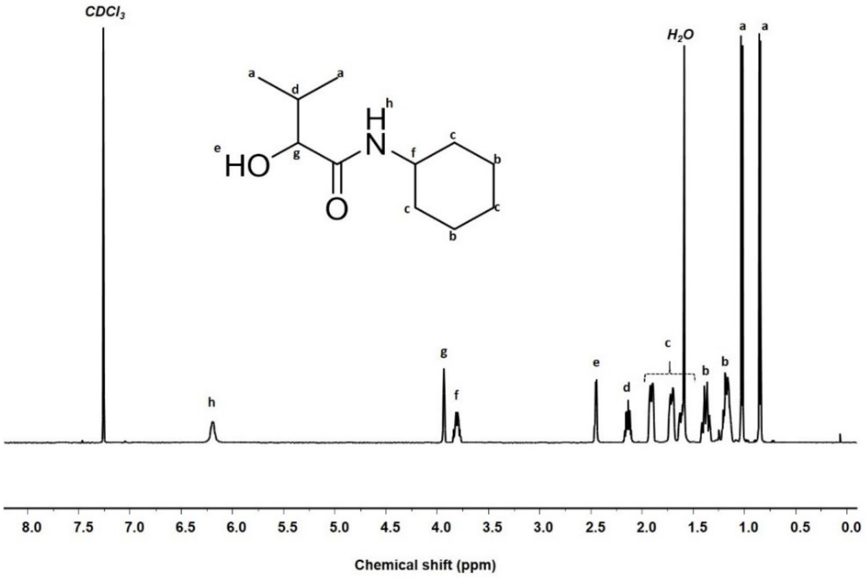

Figure SI 21a: ^{1}H ($CDCl_3$) NMR of hydrolysis product of **1c.**

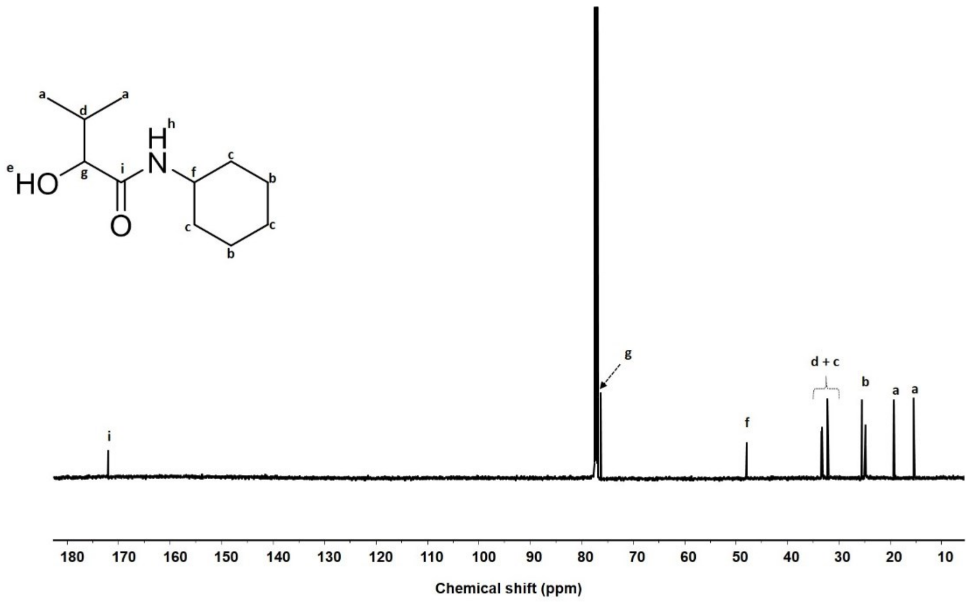

Figure SI 21b: ^{13}C ($CDCl_3$) NMR of hydrolysis product of **1c.**

Hydrolysis product molecule 3a*:* N-cyclohexyl-2-hydroxy-3-methylbutanamide

Isolated yield: 23%

^{1}H NMR (500 MHz, $CDCl_3$) δ (ppm) = 6.31 (s, NH, 1H), 3.81 (d, CH, 1H), 3.05 (s, OH, 1H), 2.09 (m, CH, 1H), 1.35 (s, 3 CH_3, 9H), 1.00, 0.83 (d, 2 CH_3, 6H).

^{13}C NMR (125 MHz, $CDCl_3$) δ (ppm) = 172.81 (h), 76.21 (e), 51.15 (g), 32.06 (c), 28.87 (b), 19.32, 15.46 (a).

ATR-IR: ν (cm^{-1}) = 3340.3, 3257.9, 2956.6, 2931.7, 2868.6, 1639.0, 1532.5, 1457.1, 1025.8, 709.9.

Exact mass: $[C_9H_{19}NO_2Na]^+$ = 196.14 $gmol^{-1}$, obtained (ESI-MS) = 196.13 $gmol^{-1}$

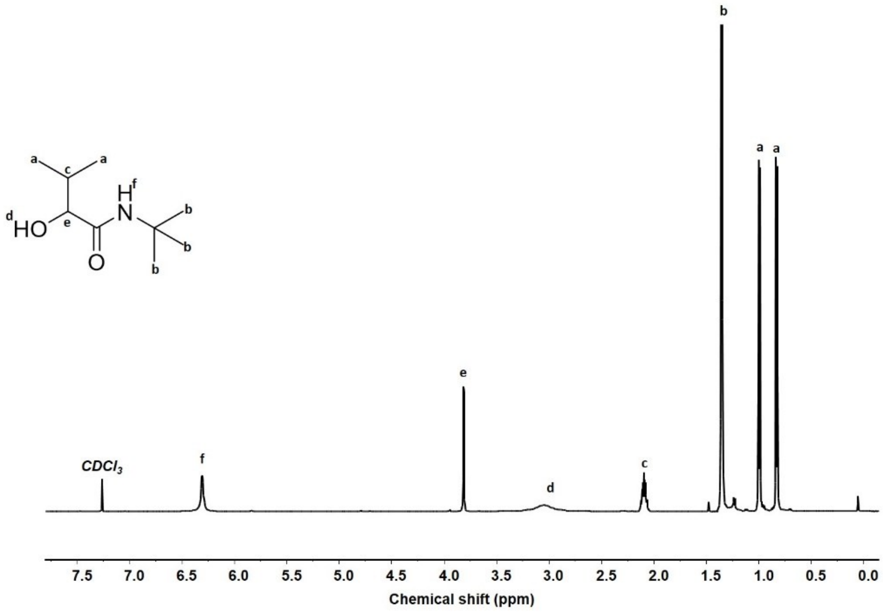

Figure SI 22a: ^{1}H ($CDCl_3$) NMR of hydrolysis product of **3a.**

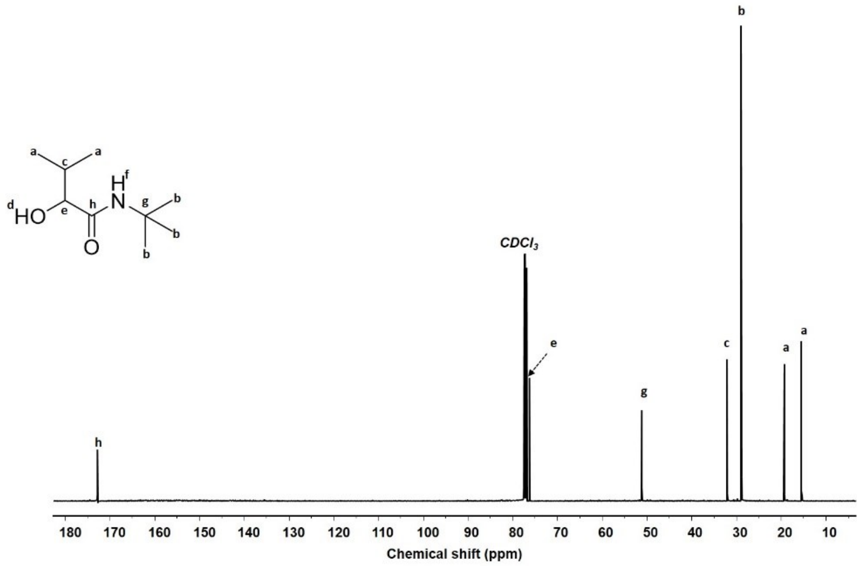

Figure SI 22b: ^{13}C ($CDCl_3$) NMR of hydrolysis product of **3a.**

Hydrolysis product molecule 9a*:* N-(tert-butyl)-2-(2,4-dinitrophenyl)-2-hydroxyacetamide

Isolated yield: 63%

^{1}H NMR (500 MHz, $CDCl_3$) δ (ppm) = 8.77 (d, Ar-CH, 1H), 8.47 (dd, Ar-CH, 1H), 7.90 (d, Ar-CH, 1H), 6.72 (s, NH, 1H), 5.61 (s, CH, 1H), 4.63 (br, OH, 1H), 1.32 (s, 3 CH_3, 9H).

^{13}C NMR (125 MHz, $CDCl_3$) δ (ppm) = 168.19 (l), 148.72 (k), 147.40 (j), 142.23 (i), 130.95 (e), 127.87 (f), 120.16 (g), 68.29 (c), 52.21 (h), 28.59 (a).

ATR-IR: ν (cm^{-1}) = 3399.0, 3294.5, 3124.0, 2927.9, 2860.6, 1655.5, 1527.5, 1347.9, 1086.9, 1060.1, 914.6, 836.2, 733.1, 710.8, 569.8.

Exact mass: $[C_{12}H_{15}N_3O_6Na]^+$ = 320.10 gmol^{-1}, obtained (ESI-MS) = 320.08 gmol^{-1}

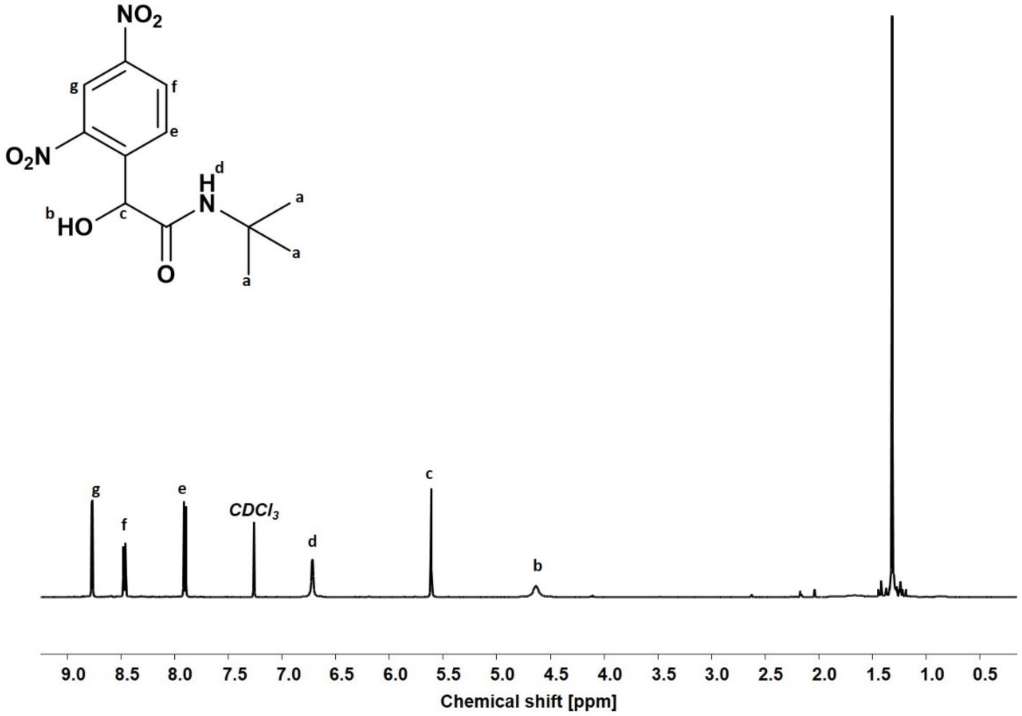

Figure SI 23a: ^{1}H ($CDCl_3$) NMR of hydrolysis product of **9a.**

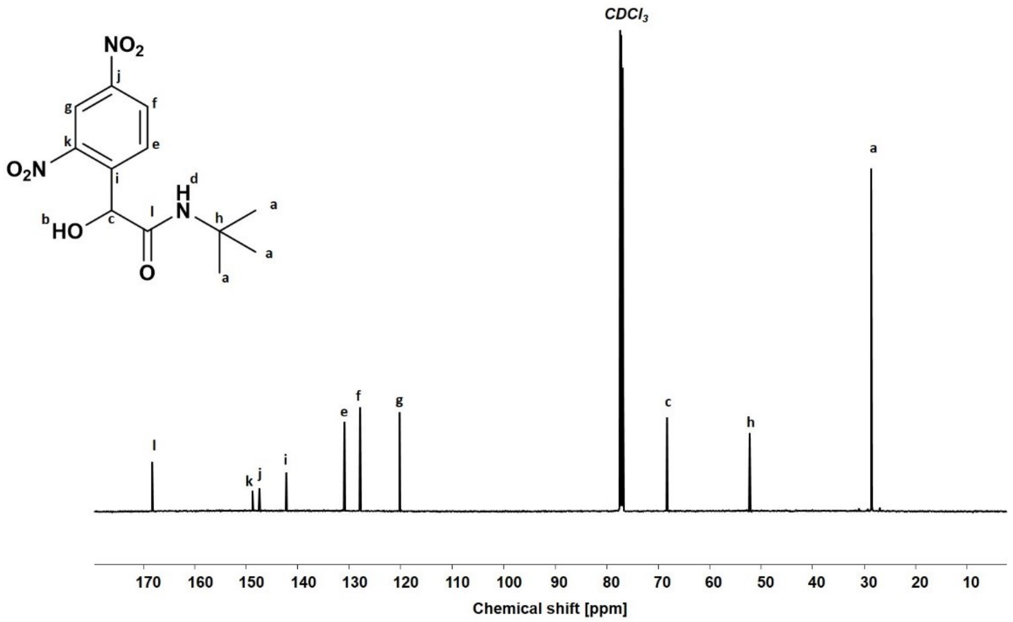

Figure SI 23b: ^{13}C ($CDCl_3$) NMR of hydrolysis product of **9a.**
